# Causes of inferior relative survival after testicular germ cell tumor diagnosed 1953–2015: A population-based prospective cohort study

**DOI:** 10.1371/journal.pone.0225942

**Published:** 2019-12-18

**Authors:** Øivind Kvammen, Tor Åge Myklebust, Arne Solberg, Bjørn Møller, Olbjørn Harald Klepp, Sophie Dorothea Fosså, Torgrim Tandstad

**Affiliations:** 1 Department of Oncology, Ålesund Hospital, Ålesund, Norway; 2 Department of Clinical and Molecular Medicine, Faculty of Medicine and Health Sciences, Norwegian University of Science and Technology, Trondheim, Norway; 3 Department of Research and Innovation, Møre and Romsdal Hospital Trust, Ålesund, Norway; 4 Department of Registration, Cancer Registry of Norway, Oslo, Norway; 5 The Cancer Clinic, St. Olav´s University Hospital, Trondheim, Norway; 6 National Advisory Unit on Late Effects after Cancer Treatment, Oslo University Hospital, The Radium Hospital, Oslo, Norway; 7 Faculty of Medicine, Oslo University, Oslo, Norway; Chang Gung Memorial Hospital at Linkou, TAIWAN

## Abstract

**Background:**

Testicular germ cell tumor (TGCT) patients and survivors have excess mortality compared to the general male population, but relative survival (RS) has been scarcely studied. We investigated causes of excess mortality and their impact on RS among men diagnosed with TGCT in Norway, 1953–2015.

**Methods and findings:**

Using registry data (n = 9541), standardized mortality ratios (SMRs) and RS were calculated. By December 31^st^, 2015, 816 testicular cancer (TC) and 1508 non-TC deaths had occurred (non-TC SMR: 1.36). Within five years of TGCT diagnosis, 80% were TC deaths. Non-TC second cancer (SC) caused 65% of excess non-TC deaths, of which 34% from gastric, pancreatic or bladder cancer. SC SMRs remained elevated ≥26 years of follow-up. In localized TGCT diagnosed >1979, SC SMRs were only elevated after seminoma. Cardiovascular disease caused 9% and other causes 26% of excess non-TC deaths, of which 58% from gastrointestinal and genitourinary disorders. RS continuously declined with follow-up. TGCT patients diagnosed >1989 had superior five-year TC-specific RS (98.3%), lower non-TC SMR (1.21), but elevated SMRs for several SCs, infections, Alzheimer’s disease, genitourinary disease and suicide. A limitation was lack of individual treatment data.

**Conclusions:**

RS declines mainly from TC deaths <5 years after TGCT diagnosis. Later, excess SC mortality becomes particularly important, reducing RS even ≥26 years. Radiotherapy; standard adjuvant seminoma treatment 1980–2007, is likely an important contributor, as are chemotherapy and possibly innate susceptibilities. Vigilant long-term follow-up, including psychosocial aspects, is important. Further research should focus on identifying survivor risk groups and optimizing treatment.

## Introduction

Despite today’s excellent cure rates for testicular germ cell tumor (TGCT), more than ten thousand men died from testicular cancer (TC) worldwide in 2012 [1]. Among TCGT survivors (TCS), excess mortality is also a concern. We previously reported a continuing decline in relative survival (RS) among men diagnosed with TGCT in Norway compared to the general male population, even beyond 25 years of follow-up [2].

TGCT treatment is associated with potentially life-threatening late effects such as second cancer (SC) and cardiovascular disease (CVD), which can manifest decades after chemo- or radiotherapy [3]. Indeed, several studies show excess mortality from these and other conditions among TCS [4–6]. However, to what extent such findings impact RS compared to the general male population is less clear.

We analyzed causes of excess mortality among TGCT patients diagnosed in Norway, 1953–2015, and examined the impact of these causes on RS.

## Methods

### Data sources

Data were obtained from the Cancer Registry of Norway (CRN) and the Norwegian Cause of Death Registry (NCDR). The study did not require institutional review board approval.

The CRN comprises data on all new cancers reported in Norway since 1953, collected prospectively. Data quality is considered to be high [7], but treatment and clinical follow-up data are incomplete. The NCDR contains cause of death information on all Norwegian inhabitants since 1951. Causes of death were recorded using the ICD-6 to ICD-9 coding systems until 1996, then ICD-10 (S1 Table).

### Study population

We included all men diagnosed with histologically verified TGCT in Norway from January 1^st^, 1953 until December 31^st^, 2015, except extragonadal germ cell tumors and spermatocytic tumors [8, 9]. Because of incomplete individual treatment data, general treatment principles at the year of diagnosis were used as a proxy (Table 1).

**Table 1:** General treatment principles for testicular germ cell tumor patients diagnosed in Norway.

Time of diagnosis	Localized disease	Metastatic disease
1953–1979	Nearly all patients received adjuvant abdominal RT to para-aortic and ipsilateral iliac lymph nodes (up to 40 and 50 Gy in seminomas and nonseminomas, respectively).	Before 1971: Large abdominal RT fields in stage II or III disease^a^. Mediastinal irradiation and/or palliative limited field RT. Chemotherapy rarely used; mainly monotherapy with cyclophosphamide or mithramycin. RPLND rarely performed. 1971 until summer of 1978: Monotherapy or combinations of cyclophosphamide, actinomycin D, doxorubicin, vincristine, or bleomycin/vinblastine, methotrexate, mithramycin. From summer of 1978: CVB, three or four courses. Bleomycin omitted if high risk of pulmonary toxicity.
1980–1989	Prophylactic mediastinal irradiation discontinued. Seminomas: adjuvant abdominal RT, dose usually 30 Gy or less. Nonseminomas: staging RPLND or, from 1989, inclusion in a surveillance program.	1980 to 86: CVB. Seminoma patients with advanced stage II disease received post-chemo RT to residual masses until 1986. RT to nonseminoma patients usually only in the palliative setting. From 1987: Transition to the BEP-regimen, three or four courses. Bleomycin omitted if high risk of pulmonary toxicity.
1990–2015	Seminomas: the usage of adjuvant RT was reduced from year 2000 and no longer considered as standard from 2007. Replaced with one course of adjuvant carboplatin. Nonseminomas: From 1995, staging RPLNDs were replaced by surveillance and adjuvant BEP.	The BEP-regimen remained standard first-line therapy. Dose-escalation to ifosfamide-containing regimens. High-dose chemotherapy with autologous stem cell support available from 1995. Stage II seminoma patients received prophylactic mediastinal RT until about year 2000. Decrease in usage of abdominal RT for stage II seminomas after year 2000, but still an option in stage 2A disease.

^a^ Stage as defined in the Royal Marsden Hospital staging system [9]

BEP, cisplatin, etoposide, bleomycin; CVB, cisplatin, vinblastine, bleomycin; Gy, Gray; RPLND, retroperitoneal lymph node dissection; RT, radiotherapy; TGCT, testicular germ cell tumor.

Patients were classified into cohorts by time period of diagnosis: 1953–1979, 1980–1989 and 1990–2015. They were further classified as either seminoma, nonseminoma or unspecified TGCT. Disease extent at diagnosis was classified by CRN variables as either localized, metastatic or unknown [10]. Nonmetastatic tumors with direct micro- or macroscopic growth into neighboring tissues were classified as localized.

Follow-up was from the time of first TGCT diagnosis until death, emigration or December 31^st^, 2015, whichever occurred first.

### Statistical analysis

In this population-based prospective cohort study, cause of death was the principal outcome parameter. Date and underlying cause of death were obtained from the NCDR for all deceased patients. Deaths were classified using the NCDR shortlist (S1 Table) by reported cause: TC, SC excluding TC, CVD and other causes including unknown (OC).

RS was computed using the method developed by Pohar Perme et al [11]. Standardized mortality ratios (SMRs) with 95% confidence intervals (CIs) were computed for all non-TC causes of death. NCDR mortality data for the general male Norwegian population, matched on 5-year age groups and calendar year, constituted the reference population. Four follow-up time subintervals were defined: <16 years, 16–<26 years, ≥26 years and ≥5 years, the latter to assess the impact of surveillance bias.

Multiple comparisons correction was not performed due to the explorative nature of the study [12, 13]. Patients with partially missing data were not included in the respective subgroup analyses. The software used was Stata/MP version 15.1, copyright 1985–2017 StataCorp LLC.

## Results

### Patient characteristics and overall mortality

In total, 9541 patients were included, of whom 5278 were diagnosed with seminoma, 4126 with nonseminoma, and 47 with an unspecified TGCT (Table 2). Overall, 79% of seminomas and 60% of nonseminomas were localized at diagnosis. Disease extent was unknown in 457 patients. Median age at diagnosis was 38 and 29 years for seminoma and nonseminoma patients, respectively. Median follow-up times were 23.5 years for TGCT patients diagnosed <1980, 28.9 years when diagnosed in the 1980s and 10.0 years when diagnosed >1989.

**Table 2:** Persons at risk, cumulative deaths and relative survival by follow-up time.

	Cohort of diagnosis	Follow-up time (years)										Total deaths at end of follow-up
		0	1	5	10	20	25	30	35	40	50	
Persons at risk (SL, SM) (NL, NM)	1953–1979	1866 (827, 236) (439, 333)	1540 (795, 162) (399, 160)	1253 (721, 115) (313, 84)	1181 (686, 102) (298, 78)	1000 (576, 74) (265, 71)	895 (510, 58) (249, 65)	771 (428, 49) (225, 56)	626 (331, 36) (201, 48)	348 (189, 17) (116, 17)	68 (32, 4) (29, 1)	
	1980–1989	1360 (530, 137) (328, 357)	1325 (527, 124) (325, 341)	1260 (510, 110) (320, 315)	1225 (493, 105) (313, 309)	1115 (434, 88) (295, 295)	1040 (389, 79) (285, 284)	547 (196, 48) (133, 168)	58 (14, 2) (20, 22)			
	1990–2015	6315 (2805, 471) (1754, 821)	5956 (2638, 436) (1658, 762)	4605 (1936, 346) (1264, 611)	3166 (1285, 247) (877, 446)	932 (416, 79) (294, 152)	135 (53, 13) (48, 21)					
Cumulative deaths (TC, SC) (CVD, OC)	1953–1979	0	325 (299, 2) (2, 22)	609 (550, 9) (14, 36)	678 (573, 22) (34, 49)	859 (593, 79) (98, 89)	962 (593, 120) (134, 110)	1086 (603, 173) (176, 134)	1228 (611, 230) (218, 169)	1356 (615, 283) (259, 199)	1492 (616, 335) (294, 247)	1518 (617, 342) (300, 259)
	1980–1989	0	35 (27, 5) (1, 2)	95 (67, 10) (10, 8)	125 (69, 18) (20, 18)	227 (71, 57) (50, 49)	298 (73, 85) (65, 75)	359 (76, 115) (74, 94)	395 (76, 135) (81, 103)			395 (76, 135) (81, 103)
	1990–2015	0	69 (41, 11) (6, 11)	189 (101, 21) (7, 50)	272 (110, 46) (35, 81)	381 (122, 84) (56, 119)	411 (123, 98) (59, 131)					411 (123, 98) (59, 131)
Relative survival, % (95% CI)	1953–1979	100	83.0 (81.2–84.6)	69.0 (66.8–71.2)	67.2 (64.7–69.6)	61.9 (58.9–64.8)	59.2 (55.8–62.4)	55.4 (51.2–59.3)	48.4 (42.4–54.1)	38.7 (32.6–44.8)	26.3 (18.3–34.9)	
	1980–1989	100	97.8 (96.8–98.5)	94.5 (92.9–95.8)	94.2 (92.2–95.7)	90.5 (87.1–93.1)	88.4 (84.5–91.4)	85.0 (79.4–89.1)				
	1990–2015	100	99.1 (98.8–99.3)	97.9 (97.3–98.3)	97.2 (96.4–97.8)	95.4 (93.3–96.9)	92.8 (88.9–95.4)					

NL or NM, nonseminoma, localized or metastatic at diagnosis; SL or SM, seminoma, localized or metastatic at diagnosis; TC, testicular cancer; SC, second cancer (excluding TC); CVD, cardiovascular disease; OC, other causes; CI, confidence interval. Cumulative deaths by histology and disease extent at diagnosis are given in S2 Table.

At end of follow-up, 2324 deaths had occurred. Of these, 816 were due to TC, 575 to SC, 440 to CVD and 493 to OC including 56 deaths of unknown cause (Table 2, S2 Table). Bilateral TC was registered in 2.9% of patients, and 102 patients had emigrated. The overall non-TC SMR was 1.36, 95% CI 1.30–1.44.

### Testicular cancer mortality

During the first five years after TGCT diagnosis, 718 of 893 deaths (80%) were caused by TC. About 90% of TC deaths occurred within five years of follow-up. TC deaths were more common in patients diagnosed <1980, among nonseminoma patients and in patients with metastatic TGCT at diagnosis (Table 2, S2 Table).

### Second cancer mortality, excluding testicular cancer

The overall SC SMR was 1.84 (95% CI 1.74–2.06), causing 262 (65%) of 402 excess non-TC deaths. Median time to SC death was 24.2 years after TGCT diagnosis (25^th^–75^th^ percentile 14.5–32.5 years).

SC SMRs ranged from 1.39 among TCS diagnosed >1989 to 2.00 among TCS diagnosed <1980 (Table 3). In general, SC SMRs increased with follow-up and were higher among TCS with metastatic TGCT at diagnosis. TCS diagnosed with localized seminoma >1979 had elevated SC SMRs, while those with localized nonseminoma did not (S3 Table).

**Table 3:** Standardized mortality ratios for selected causes of death among testicular germ cell tumor patients diagnosed in Norway.

Cause of death	Cohort of diagnosis									Code^a^
	1953–1979			1980–1989			1990–2015			
	O	SMR (95% CI)	SMR by follow-up time^b^	O	SMR (95% CI)	SMR by follow-up time	O	SMR (95% CI)	SMR by follow-up time	
**Testicular cancer**	617			76			123			
**All non-TC causes**	901	**1.42** (1.33–1.52)	A(**1.34**),B(**1.42**),C(**1.46**),D(**1.41**)	319	**1.36** (1.22–1.52)	A(**1.26**),B(**1.38**),C(**1.51**),D(**1.39**)	288	**1.21** (1.08–1.35)	A(**1.21**),D(**1.17**)	
**All non-TC second cancers**	342	**2.00** (1.79–2.23)	A(**1.70**),B(**2.12**),C(**2.05**),D(2**.03**)	135	**1.90** (1.61–2.25)	A(**1.57**),B(**1.94**),C(**2.26**),D(**1.90**)	98	**1.39** (1.15–1.70)	A(**1.27**),B(**1.77**),D(**1.45**)	2.1-TC
MN, lip, oral cavity, pharynx	3	0.99 (0.31–4.87)		5	**3.44** (1.45–10.27)	A(**5.79**),D(**2.96**)	2	1.44 (0.31–14.42)		2.1.1
MN, esophagus	7	1.99 (0.97–4.77)	C(**2.74**)	1	0.55 (–)		5	**2.61** (1.10–7.77)	B(**7.64**),D(**3.30**)	2.1.2
MN, stomach	31	**2.62** (1.86–3.81)	B(**4.29**),C(**2.64**),D(**2.89**)	13	**3.98** (2.36–7.26)	A(**4.78**),C(**4.65**),D(**4.52**)	5	1.90 (0.80–5.66)	B(**5.23**),D(**2.58**)	2.1.3
MN, colorectal, anus	44	**1.95** (1.46–2.66)	C(**2.25**),D(**2.89**)	11	1.17 (0.67–2.24)	C(**2.36**)	6	0.65 (0.30–1.71)		2.1.4
MN, liver, intrahepatic ducts	11	**5.68** (3.21–11.07)	B(**8.92**),C(**6.22**),D(**5.93**)	1	0.91 (–)		2	1.34 (0.29–13.48)		2.1.5
MN, pancreas	28	**2.97** (2.08–4.41)	B(**4.11**),C(**3.50**),D(**3.10**)	18	**4.31** (2.75–7.13)	B(**6.45**),C(**3.96**),D(**4.35**)	8	1.85 (0.95–4.16)		2.1.6
MN, trachea, bronchus, lung	47	1.25 (0.94–1.69)	A(**2.02**)	32	**1.89** (1.35–2.72)	A(**1.82**),B(**2.30**),D(**1.96**)	14	0.87 (0.53–1.55)		2.1.8
Melanoma	10	**2.53** (1.39–5.11)	B(**5.53**),D(**2.63**)	3	1.18 (0.37–5.80)		6	2.00 (0.91–5.26)		2.1.9
MN, prostate	39	**1.46** (1.08–2.04)	A(**2.43**),C(**1.53**),D(**1.42**)	7	0.83 (0.40–1.99)		9	1.30 (0.69–2.78)		2.1.14
MN, kidney	13	**2.64** (1.57–4.83)	C(**3.29**),D(**2.76**)	1	0.46 (–)		2	0.91 (0.20–9.09)		2.1.15
MN, bladder	18	**2.71** (1.73–4.48)	A(**4.03**),B(**2.96**),C(**2.32**),D(**2.64**)	10	**4.74** (2.60–9.55)	B(**3.44**),C(**10.31**),D(**5.10**)	4	2.32 (0.87–8.33)	B(**4.73**)	2.1.16
MN, brain and CNS	9	**2.06** (1.09–4.37)	B(**3.41**)	2	0.67 (0.14–6.73)		8	**1.98** (1.01–4.45)	A(**2.46**),D(**2.39**)	2.1.17
Hodgkin disease, lymphoma	6	1.32 (0.60–3.47)		1	0.44 (–)		2	0.97 (0.21–9.71)		2.1.19
Leukemia	8	1.60 (0.82–3.60)		3	1.59 (0.50–7.84)		6	**3.47** (1.59–9.15)	A(**3.83**),D(**3.04**)	2.1.20
MN, other lymph./hematol.^c^	9	**1.89** (1.00–4.00)	A(**3.91**),D(**2.16**)	2	1.65 (0.26–12.1)		1	0.71 (–)		2.1.21
MN, other (no TC deaths)	59	**3.50** (2.73–4.57)	A(**2.31**),B(**3.08**),C(**4.20**),D(**3.55**)	24	**3.68** (2.50–5.65)	A(**2.35**),B(**4.39**),C(**4.78**),D(**3.37**)	17	**2.72** (1.72–4.57)	A(**2.43**),B(**3.83**),D(**2.63**)	2.1.22
**Cardiovascular disease**	300	**1.12** (1.00–1.26)	D(**1.14**)	81	1.07 (0.85–1.34)		59	0.96 (0.75–1.26)		7.
Ischemic heart diseases	169	1.06 (0.91–1.24)		52	1.21 (0.92–1.60)		30	0.92 (0.65–1.34)		7.1
*Acute myocardial infarction*	119	1.06 (0.89–1.28)		39	1.34 (0.99–1.87)	A(**1.51**)	18	0.84 (0.54–1.40)		7.1.1
Non-ischemic heart diseases	50	**1.59** (1.20–2.13)	B(**1.86**),C(**1.55**),D(**1.59**)	16	1.42 (0.87–2.46)		13	1.25 (0.74–2.31)		7.2
Cerebrovascular diseases	44	0.88 (0.66–1.20)		5	0.37 (0.16–1.09)		13	1.20 (0.71–2.21)		7.3
Other circulatory diseases	37	**1.39** (1.02–1.96)	D(**1.40**)	8	1.02 (0.51–2.30)		3	0.45 (0.14–2.17)		7.4
**Other or unknown causes**	259	**1.34** (1.18–1.51)	A(**1.53**),C(**1.32**),D(**1.24**)	103	1.19 (0.98–1.45)	D(**1.24**)	131	**1.23** (1.04–1.46)	A(**1.25**)	
Infectious / parasitic diseases	8	1.12 (0.57–2.52)		6	1.82 (0.83–4.79)		10	**3.06** (1.69–6.18)	A(**3.64**),D(**2.47**)	1.
Endocrine, nutr., metab.^d^	15	1.50 (0.92–2.62)	B(**2.66**)	10	**2.06** (1.13–4.19)	D(**1.99**)	3	0.54 (0.17–2.64)		4.
Nervous system, sense organs	15	1.07 (0.66–1.87)		3	0.45 (0.14–2.22)		13	**1.72** (1.02–3.14)	A(**2.08**)	6.
*Alzheimer’s disease*	2	0.73 (0.16–7.26)		0	0 (–)		4	**3.85** (1.46–13.57)	A: **5.68**, D(**4.64**)	6.2
Respiratory system diseases	44	0.88 (0.66–1.20)		14	0.89 (0.55–1.56)		10	0.74 (0.41–1.50)		8.
Other respiratory diseases^e^	3	0.77 (0.24–3.79), 3	A(**4.87**)	5	**3.35** (1.41–10.00)	A(**6.80**),B(**4.63**),D(**3.48**)	1	0.71 (–)		8.4
Digestive system diseases	50	**2.83** (2.16–3.78)	B(**3.20**),C(**3.26**),D(**2.88**)	19	**2.51** (1.62–4.11)	B(**3.44**),D(**2.64**)	9	1.21 (0.64–2.57)		9.
*Ulcers*, *stomach–jejunum*	11	**3.78** (2.13–7.38)	B(**6.09**),C(**3.23**),D(**3.31**)	1	1.30 (–)		2	3.32 (0.71–33.43)		9.1
*Cirrhosis*, *fibrosis*, *c*. *hep*^f^	5	1.02 (0.43–3.03)		8	**2.57** (1.31–5.77)	A(**2.83**),D(**2.91**)	3	0.96 (0.30–4.74)		9.2
*Other digestive diseases*	34	**3.64** (2.62–5.20)	B(**3.95**),C(**4.20**),D(**3.82**)	10	**2.98** (1.62–6.05)	B(**4.40**),D(**2.89**)	4	1.24 (0.47–4.47)		9.3
Genitourinary diseases	21	**2.31** (1.54–3.64)	C(**2.70**),D(**2.50**)	2	0.87 (0.19–8.69)		7	**3.76** (1.82–8.99)	A(**5.18**),D(**2.70**)	12.
External causes	36	0.94 (0.68–1.33)		27	1.14 (0.79–1.70)		58	**1.47** (1.15–1.93)	A(**1.33**),B(**2.48**),D(**1.49**)	17.
*Accidents*	25	0.90 (0.62–1.38)		17	1.15 (0.73–1.93)		35	**1.46** (1.06–2.07)	B(**2.59**),D(**1.53**)	17.1
*Suicide*	10	1.02 (0.56–2.08)		10	1.23 (0.68–2.49)		22	**1.54** (1.03–2.42)		17.2

Causes of death are classified according to S1 Table. Statistically significant results (P = <0.05) are highlighted in bold.

CI, confidence interval; O, observed deaths in the study population; MN, malignant neoplasm; SMR, standardized mortality ratio; TC, testicular cancer.

^a^ Code for cause of death as defined in S1 Table.

^b^ Subgroups with statistically significant SMRs pertaining to follow-up time, given in parentheses: A, <16 years follow-up only; B, 16-<26 years follow-up only; C, ≥26 years follow-up only; D, ≥5 years follow-up only. SMRs for additional conditions with 95% CI by follow-up, histology and disease extent at diagnosis are given in S3 Table.

^c^ Other malignant neoplasms of lymphoid and hematopoietic tissue.

^d^ Endocrine, nutritional and metabolic diseases.

^e^ Excluding influenza (code 8.1), pneumonia (code 8.2) and chronic lower respiratory diseases (code 8.3).

^f^ Chronic hepatitis.

Gastrointestinal and non-TC genitourinary cancer caused 38% and 15% of excess SC deaths, respectively. Gastric, pancreatic and bladder cancer caused 34% of excess SC deaths combined, with SMRs of 2.62–2.97 in TCS diagnosed <1980 and 3.98–4.74 among those diagnosed in the 1980s. For gastric and bladder cancers, SMRs were also elevated ≥16 years of follow-up among TGCT patients diagnosed >1989 (Table 3).

SMRs were generally threefold elevated in the “other malignant neoplasms” group. This group comprises several cancer forms, for which separate SMRs were not calculated (S1 Table). However, about half of deaths in this group were due to either sarcoma or cancer of unknown origin.

TCS diagnosed <1980 also had elevated SMRs for cancer of the large intestine, liver or intrahepatic bile ducts, prostate and central nervous system, melanoma, certain hematological malignancies, and esophageal cancer (the latter ≥26 years of follow-up only). Additionally, there was an about twofold risk of death from cancer of the lung, trachea or bronchus <16 years of follow-up.

TCS diagnosed in the 1980s had an SMR of 1.89 for cancer of the lung, trachea or bronchus. The SMR for cancer of the lip, oral cavity or pharynx was 3.44. ≥26 years of follow-up, there was an about twofold risk of death from cancer of the large intestine.

Among TCS diagnosed >1989, the SMRs for leukemia, esophageal and central nervous system cancer were 3.47, 2.61 and 1.98, respectively.

### Cardiovascular disease mortality

The CVD SMR for all TCS was borderline significant at 1.09 (95% CI 0.99–1.22), causing 35 (9%) of excess non-TC deaths. Median time to CVD death was 21.4 years (25^th^–75^th^ percentile 12.2–32.4 years). Thirty-one (89%) of the excess CVD deaths, mostly non-ischemic heart diseases, occurred among TCS initially diagnosed with metastatic TGCT.

Among TCS diagnosed <1980, the overall CVD SMR was 1.12, and 1.88 for TCS diagnosed with metastatic seminoma. The SMR for non-ischemic heart diseases was 1.59 (Table 3 and S3 Table).

TCS diagnosed in the 1980s had a 50% increased risk of death from acute myocardial infarction <16 years of follow-up, as had TCS diagnosed with localized seminoma (S3 Table). TCS diagnosed with metastatic seminoma had an about fivefold risk of death from non-ischemic heart diseases.

The only significant finding in TCS diagnosed >1989 was among TCS diagnosed with metastatic nonseminoma with <16 years of follow-up, where the CVD SMR was 2.23 (S3 Table). Five of seven deaths were due to heart diseases.

### Other cause mortality

The OC SMR for all TGCT patients was 1.27 (95% CI 1.17–1.39), causing 105 (26%) of 402 excess non-TC deaths. Of these, 18 (17%) were of unknown cause (Table 3, S3 Table). Median time to OC death was 19.1 years (25^th^–75^th^ percentile 7.7–31.0 years).

Digestive and genitourinary diseases caused 58% of excess deaths in the OC category. SMRs for digestive diseases were increased about threefold in TCS diagnosed <1990 (Table 3). Thirteen of 45 excess deaths by digestive diseases were due to ulcers or chronic liver disease, while intestinal disorders caused most of the remaining excess deaths.

Among TCS diagnosed <1980, the SMR for genitourinary diseases was 2.31. TCS diagnosed in the 1980s had a twofold risk of death from endocrine, nutritional and/or metabolic diseases and a threefold risk of death from a subgroup of respiratory disorders not including pneumonia and chronic lower respiratory disorders (S1 Table).

TCS diagnosed >1989 had elevated SMRs for genitourinary diseases (3.76) including diseases of the kidney and ureter (3.18), infections (3.06) and nervous system / sense organ diseases (1.72) including Alzheimer’s disease (3.85). Elevated SMRs for suicide (1.54) and accidents (1.46) were found, elevated also among nonseminoma patients with metastases at diagnosis (S3 Table). Median time to suicide among TCS diagnosed >1989 was 7.1 years (25^th^–75^th^ percentile 2.8–12.7 years), and the median age at suicide was 40.1 years (25^th^–75^th^ percentile 34.0–50.0 years).

### Mortality among five-year TGCT survivors

In general, restricting SMR analyses to the 7111 five-year TCS caused only minor changes in SMRs from those of the entire study population (Table 3, S3 Table). Notable exceptions were that the SMRs for suicide and nervous system diseases in TCS diagnosed >1989 were no longer significantly elevated. This was also true for central nervous system cancer in TCS diagnosed <1980. Conversely, the SMR for stomach cancer became significantly elevated for five-year TCS diagnosed >1989, bladder cancer for TCS diagnosed <1980, as did the OC SMR among TCS diagnosed in the 1980s.

### Relative survival by cause of death category, all TGCT patients

**Fig 1:**
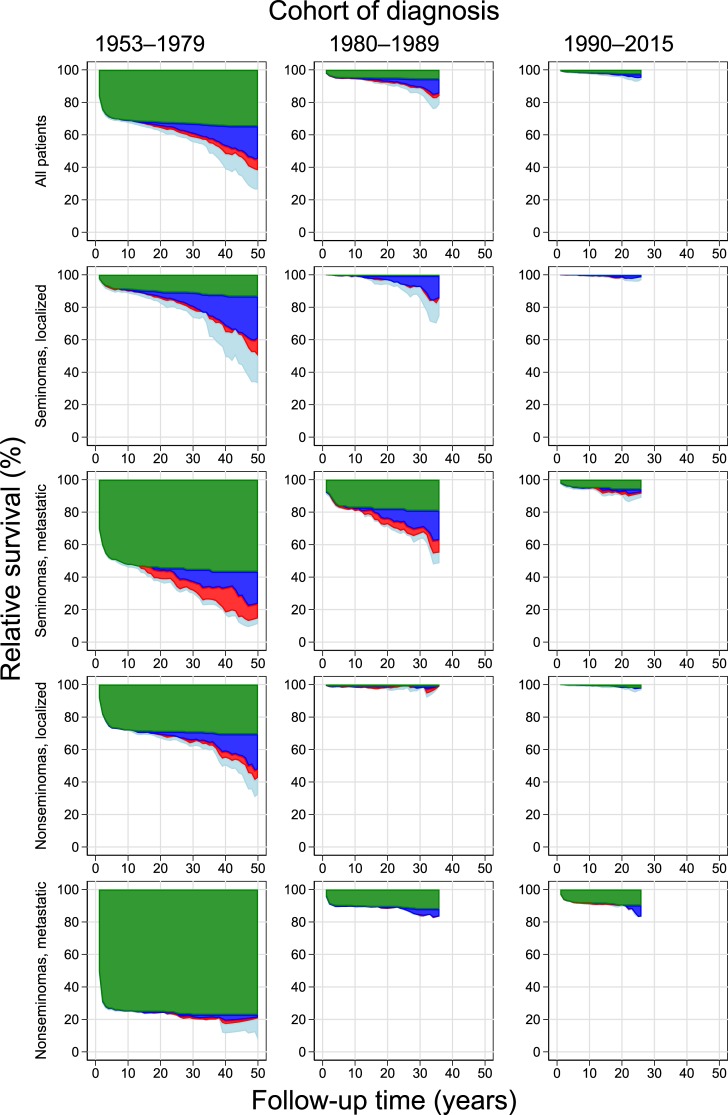
Point estimates for relative survival among testicular germ cell patients diagnosed in Norway, by histology and disease extent at diagnosis, with cause of death category. Survival in the reference population is always 100%.

RS among TGCT patients generally declined with increasing follow-up time (Fig 1, Table 2). While TC deaths were the main cause of reduced RS during the first five years of follow-up, non-TC causes gradually became dominant beyond this time, with elevated SMRs among TCS increasing with follow-up even ≥26 years. Overall, SC was the prime non-TC contributor to reduced RS (Fig 1, Table 3). Patients diagnosed with localized seminoma >1979 had increased overall non-TC SC SMRs, while patients with localized nonseminoma did not, contributing to the inferior RS point estimates for this patient group.

Both short- and long-term RS improved significantly from TGCT patients diagnosed <1980 to patients diagnosed in the 1980s. Five-year RS increased from 69.0 to 94.5%, while 25-year RS increased from 59.2 to 88.4%. Further increases in RS point estimates were seen among TGCT patients diagnosed >1989 (Fig 1, Table 2). Five-year TC-specific RS increased from 70.1% (95% CI 68.0–72.1%) among patients diagnosed <1980 to 95.0% (95% CI 93.7–96.1%) in the 1980s and 98.3% (95% CI 97.9–98.6%) >1989.

## Discussion

The primary objective of this study was to give an overview of causes of excess mortality and their impact on RS among men diagnosed with TGCT in Norway, 1953–2015, compared with the general Norwegian male population.

TC was, unsurprisingly, the main cause of declining RS during the first five years after diagnosis. Non-TC SC became the prime contributor to the continuing decline in RS beyond this time, particularly due to excess mortality from gastrointestinal and non-TC genitourinary cancer. Similarly, non-malignant digestive and genitourinary diseases were important contributors to excess OC mortality. CVD was a comparatively minor contributor, with most excess deaths occurring among TCS diagnosed with metastatic TGCT.

Other notable findings included the elevated non-TC SMRs among seminoma patients diagnosed with localized disease >1979, and the elevated SMR for suicide in patients diagnosed >1989.

Increased SMRs for central nervous system cancer and nervous / sensory system diseases including Alzheimer’s disease, were novel findings, though based on few cases. Many studies have shown an inverse relationship between cancer and dementia, although bias cannot be ruled out [14]. Prostate cancer excess mortality among TCS diagnosed <1980 was also a novel finding, though consistent with excess risks previously reported [15, 16]. Patients with a previous genitourinary cancer are perhaps more likely to be screened for prostate cancer, thus increasing detection.

To our knowledge, this is the first study to examine the impact of any cause of excess mortality on RS among TGCT patients. Study strengths are the inclusion of almost ten thousand patients diagnosed both in the pre- and post-cisplatin era regardless of disease extent and histology, inclusion of all causes of death, and the long follow-up times, all using high quality data sources. We believe our study has high external validity.

Several studies have reported long-term cause-specific mortality data among TC patients (S4 Table). Most of these studies are registry based and lack complete treatment data.

### Mortality after radiotherapy

Radiation-induced DNA damage may lead to long-term effects such as stromal change with collagen deposition and neoangiogenesis causing organ dysfunction [17]. An increased SC risk within radiation fields with a dose-response relationship has been reported among TCS [18]. Increased mortality from SC, CVD, gastrointestinal diseases and infections has also been reported after radiotherapy (S4 Table). Zagars et al reported an SC SMR of 1.91 and a CVD SMR of 1.61 beyond 15 years of follow-up among stage I-II seminoma patients. Prophylactic mediastinal irradiation was the only factor correlated with survival in univariate analysis [19].

In Norway, most patients diagnosed in the pre-cisplatin era received infradiaphragmatic radiotherapy. Adjuvant radiotherapy was omitted in nonseminoma patients from 1980, whereas stage I seminoma patients continued to receive adjuvant irradiation to the paraaortic lymph nodes until about year 2007. Stage II seminoma patients received prophylactic mediastinal radiotherapy until about year 2000 (Table 1).

Thus, our findings suggest that infradiaphragmatic radiotherapy is a strong contributor to declining long-term RS by excess mortality from SC and OC in TCS diagnosed <1980, as well as in patients with localized seminoma. Mediastinal radiotherapy may likewise explain the excess CVD mortality among patients with metastatic seminoma diagnosed <1980, or the excess lung cancer mortality among corresponding patients diagnosed in the 1980s.

### Mortality after chemotherapy

Cisplatin-based chemotherapy regimens are associated with a wide range of toxicities and late effects, including nephrotoxicity, CVD and SC [20–22]. Such treatment might increase the CVD risk directly through vascular damage, or indirectly through modifying CVD risk factors, such as obesity, hypercholesteremia and hypertension [23]. Cisplatin can be detected in the blood and urine for decades after treatment, and serum levels have been positively correlated to SC risk [21, 24]. Cisplatin and etoposide have been linked to excess leukemia risk, often manifesting earlier than solid cancers [25]. Bleomycin can cause life-threatening pulmonary toxicity [20].

Several studies have analyzed long-term mortality among TGCT patients who received chemotherapy (S4 Table). Kier et al [26] reported a 1.6 times risk of SC death among Danish patients diagnosed between 1984 and 2008. Fung et al [5] reported a CVD SMR of 1.36 among nonseminoma patients diagnosed between 1980 and 2010.

Combined radio- and chemotherapy yields a higher risk of non-TC death than either treatment alone. Conversely, patients having undergone initial surgery only seem to be at lower risk [26]. Our findings of excess mortality from CVD, SC, respiratory and genitourinary diseases could thus partly be chemotherapy-related.

### Treatment-independent mortality

SC has been reported to be more common among seminoma patients [15, 16, 27], who are approximately ten years older than nonseminoma patients at diagnosis, possibly causing a reduced long-term tolerance to treatment [28]. Moreover, previous TCGT treatment may hamper the possibility to provide effective SC treatment [29]. Repeated CT scans could be associated with elevated SC risks [30].

TC development occurs by an interaction between polygenetic, environmental and hormonal causes [31]. TGCT patients might genetically be more susceptible to developing life-threatening diseases such as cancer. A recent study on TC patients diagnosed 1980–2009 in Norway with complete treatment information, found increased SC risk even after surgery only [32].

Increased suicide risk has been reported among US TCS [33–35]. In a recent Norwegian study, TCS born between 1965 and 1985, diagnosed before age 25, had a suicide hazard ratio of 2.9 [36].

Some evidence suggests increased prevalence of anxiety disorder [37] and fatigue [38] among TC patients. In a 2016 study, increased prevalence of depression and reduced health-related quality of life was also found [39], though other studies indicate that health-related quality of life in TCS is similar to the general population [40]. These findings could partly explain the increased suicide risk, as could changes in coding practices.

Conversely, general health care advances during the last decades have probably improved survival in the study population as well.

### Study limitations

Incomplete CRN treatment and relapse data makes the long-term effects of a particular treatment difficult to assess.

Potential differences in comorbidity and smoking habits, for which we had no data, could affect SMRs and RS [41]. For instance, men with Down’s syndrome have increased risks for both TC and several other conditions such as Alzheimer’s disease [42]. With an overall median age of <40 years at diagnosis, we nevertheless expect little pre-TGCT comorbidity, and smoking habits were likely similar in the reference population [43].

TC deaths were not excluded from the NCDR reference population data, which could have led to a slight underestimation of overall SC SMRs. SMR and RS subgroup comparisons should be interpreted with caution due to potential differences in age distribution, follow-up time and reference population mortality rate. The decision to not perform multiple comparisons correction increases the risk of type I errors. Several SMRs are based on a relatively low number of cases, but there was no way of expanding the study sample as all eligible patients were included.

Surveillance bias must be considered, particularly during the first five years of follow-up. It is possible, for instance, that a patient that has previously been diagnosed with cancer is more likely to have any subsequent condition detected due to more vigorous follow-up. This could ultimately have an impact on survival. We included TGCT patients followed for <5 years in the analyses as to not infer a particular mechanism behind excess deaths (i.e., treatment-induced as opposed to genetic or other conditions), but also to provide the most complete estimate of RS across the entire follow-up period from time of TGCT diagnosis.

To investigate the extent of surveillance bias, we performed separate SMR analyses on five-year TCS only (Table 3, S3 Table). Because of the long MTD for most conditions, it was as expected that most SMRs did not significantly change for analyses restricted to five-year survivors. A few SMRs were no longer significantly elevated, perhaps most notably the important finding of increased suicide risk among TCS diagnosed >1989. As the MTD was 7.1 years and the number of cases was limited, such a result was not unexpected. The SMR point estimate of 1.54 remained unchanged. We conclude that the overall impact of surveillance bias on our results is negligible.

## Conclusions

Despite the improved prognosis for cure, death by TC remains the main cause of excess mortality the first five years of follow-up among TGCT patients diagnosed in Norway. TCS also remain at increased long-term risk of death by SC in particular, negatively impacting RS even beyond 25 years of follow-up. Malignant and non-malignant diseases of the gastrointestinal and genitourinary organs are among the main long-term causes of excess mortality, while CVD is a comparatively minor cause. Late effects of radio- and chemotherapy are the main culprits. The elevated non-TC SMRs among seminoma patients diagnosed in the 1980s could be due to radiotherapy given in early-stage disease.

RS point estimates are highest among patients diagnosed >1989, but follow-up time is also the shortest. Excess mortality among these patients, including suicide, is a concern. Continuing optimization of TGCT treatment and appropriate follow-up schemes are thus required, covering psychosocial health as well. Particular focus should be on the follow-up of patients previously treated with radio- and/or chemotherapy. Further research should also be directed towards identifying subgroups of TGCT patients and survivors at particular risk of excess mortality.

## Supporting information

S1 Table Extended EU shortlist for causes of death, in use by the Norwegian Cause of Death Registry. (DOCX)

S2 Table Cumulative deaths by cause of death, histology, disease extent at diagnosis and follow-up time. (DOCX)

S3 Table Standardized mortality ratios for selected causes of death among testicular germ cell tumor patients diagnosed in Norway. (DOCX)

S4 Table Summary of selected publications reporting long-term cause-specific mortality data among testicular cancer patients. (DOCX)

S5 Table STROBE checklist. (DOC)

## Abbreviations

CI: confidence interval
CRN: Cancer Registry of Norway
CVD: cardiovascular disease
NCDR: Norwegian Cause of Death Registry
OC: other causes
RS: relative survival
SC: second cancer
SMR: standardized mortality ratio
TC: testicular cancer
TCS: testicular cancer survivors
TGCT: testicular germ cell tumor
