# Supplementary material for: Causes of inferior relative survival after testicular germ cell tumor diagnosed 1953–2015: A population-based prospective cohort study
Source: PLoS One. 2019 Dec 18;14(12):e0225942. doi: 10.1371/journal.pone.0225942 (PMC6919610; doi:10.1371/journal.pone.0225942)
Supplement: S1 Table — (DOCX) [file pone.0225942.s001.docx]

| **S1 Table. Extended European shortlist for causes of death, in use by the Norwegian Cause of Death Registry.** | | | | | |
| --- | --- | --- | --- | --- | --- |
| **Code** | **Description** | **ICD-10** | **ICD-9** | **ICD-8** | **ICD-6/7** |
| 1. | Infectious and parasitic diseases | A00-B99 | 001-139 | 000-136 | 001-138, 571 |
| 1.1 | Tuberculosis | A15-A19, B90 | 010-018, 137 | 010-019 | 001-019 |
| 1.2 | AIDS (HIV-disease) | B20-B24 | 042-044 (279.1) | - | - |
| 1.3 | Viral hepatitis | B15-B19, B94.2 | 070 | 070 | 092 |
| 1.4 | Other infectious and parasitic diseases | A00-A09,  A20-B09,  B25-B89,  B91-B94.1, B94.8-B99 | 001-009,  020-041,  045-066,  071-136,  138-139 | 000-009,  020-068,  071-136 | 020-091,  093-138,  571 |
| 2. | Neoplasms | C00-D48 | 140-239 | 140-239 | 140-239 |
| 2.1* | Malignant neoplasms | C00-C97 | 140-208 | 140-209 | 140-207 |
| 2.1.1 | Malignant neoplasm of lip, oral cavity, pharynx | C00-C14 | 140-149 | 140-149 | 140-148 |
| 2.1.2 | Malignant neoplasm of esophagus | C15 | 150 | 150 | 150 |
| 2.1.3 | Malignant neoplasm of stomach | C16 | 151 | 151 | 151 |
| 2.1.4 | Malignant neoplasm of colon, rectum and anus | C18-C21 | 153-154 | 153-154 | 153-154 |
| 2.1.5 | Malignant neoplasm of liver and intrahepatic bile ducts | C22 | 155 | 155, 197.8 | 155-156 |
| 2.1.6 | Malignant neoplasm of pancreas | C25 | 157 | 157 | 157 |
| 2.1.7 | Malignant neoplasm of larynx | C32 | 161 | 161 | 161 |
| 2.1.8 | Malignant neoplasm of trachea, bronchus, lung | C33-C34 | 162 | 162 | 162-163 |
| 2.1.9 | Malignant melanoma of skin | C43 | 172 | 172 | 190 |
| 2.1.10 | Malignant neoplasm of breast | C50 | 174-175 | 174 | 170 |
| 2.1.11 | Malignant neoplasm of cervix uteri | C53 | 180 | 180 | 171 |
| 2.1.12 | Malignant neoplasm of other and unspecified parts of uterus | C54-C55 | 179, 182 | 182 | 172, 174 |
| 2.1.13 | Malignant neoplasm of ovary | C56 | 183.0 | 183.0 | 175 |
| 2.1.14 | Malignant neoplasm of prostate | C61 | 185 | 185 | 177 |
| 2.1.15 | Malignant neoplasm of kidney | C64 | 189.0 | 189.0 | 180 |
| 2.1.16 | Malignant neoplasm of bladder | C67 | 188 | 188 | 181 |
| 2.1.17 | Malignant neoplasm of brain and central nervous system | C70-C72 | 191-192 | 191-192 | 193.0-193.2 |
| 2.1.18 | Malignant neoplasm of thyroid | C73 | 193 | 193 | 194 |
| 2.1.19 | Hodgkin disease and lymphomas | C81-C86 | 200-201 | 200-201 | 201-202 |
| 2.1.20 | Leukemia | C91-C95 | 204-208 | 204-208 | 204 |
| 2.1.21 | Other malignant neoplasms of lymphoid and hematopoietic tissue | C88, C90, C96 | 202-203 | 202-203 | 200, 203, 205, 206, 207 |
| 2.1.22^a^ | Other malignant neoplasms | C17, C23-C24, C26-C31, C37-C41, C44-C49, C51-C52, C57-C60, **C62**-C63, C65-C66, C68-C69, C74-C80, C97 | 152, 156,  158-160,  163-171,  173, 181,  183.2-184,  **186**-187,  189.1-190,  194-199 | 152, 156,  158-160,  163-171,  173, 181,  183.1-184,  **186**-187,  189.1-190,  194-197.7,  197.9-199 | 152, 158-  160, 164-  165, 173,  176, **178**-  179, 191-  192, 193.3-  193.9, 195-  199 |
| 2.2 | Non-malignant neoplasms (benign and uncertain) | D00-D48 | 209-239 | 210-239 | 210-239 |
| 3. | Diseases of the blood and blood forming organs and certain disorders involving the immune mechanism | D50-D89 | 280-289 | 280-289 | 290-299 |
| 4. | Endocrine, nutritional and metabolic diseases | E00-E89 | 240-279 | 240-279 | 250-289 |
| 4.1 | Diabetes mellitus | E10-E14 | 250 | 250 | 260 |
| 4.2 | Other endocrine, nutritional and metabolic diseases | E00-E07, E15-E89 | 240-246, 251-279 | 240-246, 251-279 | 250-254, 270-289 |
| 5. | Mental and behavioral disorders | F01-F99 | 290-319 | 290-315 | 300-326 |
| 5.1 | Dementia | F01, F03 | 290 | 290 | 304-305 |
| 5.2 | Alcohol abuse (including alcoholic psychosis) | F10* | 291, 303 | 291, 303 | 307, 322 |
| 5.3 | Drug dependence, toxicomania | F11*-F16*, F18*-F19* | 304-305 | 304-305 | 323 |
| 5.4 | Other mental and behavioral disorders | F04-F09, F17*, F20-F99 | 292-302, 306-319 | 292-302, 306-315 | 300-306, 308-321, 324-326 |
| 6. | Diseases of the nervous system and the sense organs | G00-H95 | 320-389 | 320-389 | 340-398 |
| 6.1 | Parkinson’s disease | G20 | 332.0 | 342 | 350 |
| 6.2 | Alzheimer’s disease | G30 | 331.0 | - | - |
| 6.3 | Other diseases of the nervous system and the sense organs | G00-G12, G14, G21-G25, G31-H95 | 320-330, 331.1-331.9, 332.1-389 | 320-341, 343-389 | 340-345, 351-398 |
| 7. | Diseases of the circulatory system | I00-I99 | 390-459 | 390-444.1, 444.3-458, 782.4 | 330-334, 400-468, 782.4 |
| 7.1 | Ischemic heart diseases | I20-I25 | 410-414 | 410-414 | 420, 422.1 |
| 7.1.1 | Acute myocardial infarction | I21-I22 | 410-411 | 410-411 | 420.1 |
| 7.1.2 | Other ischemic heart diseases | I20, I23-I25 | 412-414 | 412-414 | 420.0, 420.2, 422.1 |
| 7.2 | Other heart diseases | I30-I51 | 420-429 | 420-429 | 430-434 |
| 7.3 | Cerebrovascular diseases | I60-I69 | 430-438 | 430-438 | 330-334 |
| 7.4 | Other diseases of the circulatory system | I00-I15, I26-I28, I70-I99 | 390-405, 415-417, 440-459 | 390-404, 440-444.1, 444.3-458, 782.4 | 400-416, 421, 422.0, 422.2, 440-468, 782.4 |
| 8. | Diseases of the respiratory system | J00-J99 | 460-519 | 460-519 | 470-527, 240, 241 |
| 8.1 | Influenza | J09-J11 | 487 | 470-474 | 480-493 |
| 8.2 | Pneumonia | J12-J18 | 480-486 | 480-486 | 490-493 |
| 8.3 | Chronic lower respiratory diseases | J40-J47 | 490-494, 496 | 491-493, 518 | 241, 501, 502, 526, 527.1 |
| 8.3.1 | Asthma | J45-J46 | 493 | 493 | 241 |
| 8.3.2 | Other chronic lower respiratory diseases | J40-J44, J47 | 490-492, 494, 496 | 491-492, 518 | 501, 502, 526, 527.1 |
| 8.4 | Other diseases of the respiratory system | J00-J06, J20-J39, J60-J99 | 460-478, 495, 500-519 | 460-466, 490, 500-517, 519 | 240, 470-475, 500, 510-525, 527.0, 527.2 |
| 9. | Diseases of the digestive system | K00-K92 | 520-579 | 520-577, 444.2 | 530-570, 572-587 |
| 9.1 | Ulcer of stomach, duodenum and jejunum | K25-K28 | 531-534 | 531-534 | 540-542 |
| 9.2 | Cirrhosis, fibrosis and chronic hepatitis | K70, K73-K74 | 571 | 571 | 581 |
| 9.3 | Other diseases of the digestive system | K00-K22, K29-K66, K71-K72, K75-K92 | 520-530, 535-570, 572-579 | 520-530, 535-570, 572-577, 444.2 | 530-539, 543-570, 572-580, 582-587 |
| 10. | Diseases of the skin and subcutaneous tissue | L00-L99 | 680-709 | 680-709 | 690-716 |
| 11. | Diseases of the musculoskeletal system / connective tissue | M00-M99 | 710-739 | 710-738 | 720-749 |
| 11.1 | Rheumatoid arthritis and osteoarthrosis | M05-M06, M15-M19 | 714-715 | 712-713 | 722-723 |
| 11.2 | Other diseases of the musculoskeletal system / connective tissue | M00-M02, M08-M13, M20-M99 | 710-712, 716-739 | 710-711, 714-738 | 720-721, 724-749 |
| 12. | Diseases of the genitourinary system | N00-N99 | 580-629 | 580-629, 792 | 590-637, 792 |
| 12.1 | Diseases of kidney and ureter | N00-N29 | 580-594 | 580-594 | 590-604 |
| 12.2 | Other diseases of the genitourinary system | N30-N99 | 595-629 | 595-629, 792 | 605-637, 792 |
| 13. | Complications of pregnancy, childbirth and puerperium | O00-O99 | 630-676 | 630-678 | 640-689 |
| 14. | Certain conditions originating in the perinatal period | P00-P96 | 760-779 | 760-779 | 760-777 |
| 15. | Congenital malformations and chromosomal abnormalities | Q00-Q99 | 740-759 | 740-759 | 750-759 |
| 16. | Symptoms, signs, ill-defined causes | R00-R99 | 780-799 | 780-782.3, 782.5-791, 793-796 | 780-782.3, 782.5-791, 793-795 |
| 16.1 | Sudden infant death syndrome | R95 | 798.0 | - | - |
| 16.2 | Unknown and unspecified causes | R96-R99 | 798.1-9, 799.0,2-3,5-9 | 796-796 | 795 |
| 16.3 | Other symptoms, signs, ill-defined causes | R00-R94 | 780-797, 799.1, 799.4 | 780-782.3, 782.5-791, 793-794 | 240, 242-245, 780-782.3, 782.5-791, 793-794 |
| 17. | External causes of morbidity and mortality | V01-Y89 | E800-E999 | E800-E999 | E800-E999 |
| 17.1 | Accidents | V01-X59, Y85-Y86 | E800-E929 | E800-E929, E940-E946 | E800-E936, E960-E962 |
| 17.1.1 | Transport accidents | V01-V99, Y85 | E800-E845, E929.0-1 | E800-E845, E940-E941 | E800-E866 |
| 17.1.2 | Accidental falls | W00-W19 | E880-E888 | E880-E887 | E900-E904 |
| 17.1.3 | Drowning and accidental submersion | W65-W74 | E910 | E910 | E929 |
| 17.1.4 | Accidental poisoning | X40-X49 | E850-E869 | E850-E877 | E870-E895 |
| 17.1.5 | Other accidents | W20-W64, W75-X39, X50-X59, Y86 | E870-E879, E890-E909, E911-E928, E929.2-9 | E890-E909, E911-E929, E942-E946 | E910-E928, E930-E936, E960-E962 |
| 17.2 | Suicide and intentional self-harm | X60-X84, Y87.0 | E950-E959 | E950-E959 | E963, E970-E979 |
| 17.3 | Homicide, assault | X85-Y09, Y87.1 | E960-E969 | E960-E969 | E980-E983, E964 |
| 17.4 | Events of undetermined intent | Y10-Y34, Y87.2 | E980-E989 | E980-E989 | - |
| 17.5 | Other external causes of injury and poisoning | Y35-Y84, Y88-Y89 | E930-E949, E970-E978, E990-E999 | E930-E936, E943-E949, E970-E978, E990-E999 | E940-E959, E965, E984, E990-E999 |
| ICD: International Classification of Diseases  From reference year 2006, ICD-10 codes from F10 to F19 having the 4th digit coded ‘0’ (acute intoxication) are recoded to ICD-10 codes X41, X42, X44, X45, X46 or X49 with the 4^th^ digit coded ‘9’, according to the 2006 update of the ICD-10 classification. The table is an extension of the European Shortlist for Causes of Death, May 2012 [1].  ^a^ The highlighted ICD-codes under code 2.1.22 were used in this study to identify deaths by testicular cancer, and to exclude from analyses as appropriate. | | | | | |

**References, S1 Table**

1. Eurostat. European Shortlist of Causes of Death, May 2012. Available from: <http://ec.europa.eu/eurostat/ramon/nomenclatures/index.cfm?TargetUrl=LST_NOM_DTL&StrNom=COD_2012&StrLanguageCode=EN&IntPcKey=&StrLayoutCode=HIERARCHIC>.
