# Supplementary material for: Causes of inferior relative survival after testicular germ cell tumor diagnosed 1953–2015: A population-based prospective cohort study
Source: PLoS One. 2019 Dec 18;14(12):e0225942. doi: 10.1371/journal.pone.0225942 (PMC6919610; doi:10.1371/journal.pone.0225942)
Supplement: S2 Table — (DOCX) [file pone.0225942.s002.docx]

| **S2 Table. Cumulative deaths by cause of death, histology, disease extent at diagnosis and follow-up time.** | | | | | | | | | | | | |
| --- | --- | --- | --- | --- | --- | --- | --- | --- | --- | --- | --- | --- |
|  | **Cohort of diagnosis** | **Follow-up time (years)** | | | | | | | | | | **Total deaths at end of follow-up** |
|  |  | **0** | **1** | **5** | **10** | **20** | **25** | **30** | **35** | **40** | **50** |  |
| Cumulative TC deaths  (SL, SM)  (NL, NM) | 1953–1979 | 0 | 299  (20, 71)  (36, 165) | 550  (69, 115)  (116, 239) | 573  (73, 122)  (122, 244) | 593  (84, 126)  (126, 245) | 593  (86, 127)  (126, 248) | 603  (87, 127)  (127, 250) | 611  (93, 127)  (128, 250) | 615  (94, 128)  (130, 250) | 616  (95, 128)  (130, 250) | 617  (95, 128)  (131, 250) |
|  | 1980–1989 | 0 | 27  (1, 10)  (2, 14) | 67  (4, 22)  (4, 36) | 69  (4, 23)  (4, 37) | 71  (4, 24)  (4, 38) | 73  (4, 24)  (4, 40) | 76  (4, 25)  (4, 42) | 76  (4, 25)  (4, 42) |  |  | 76  (4, 25)  (4, 42) |
|  | 1990–2015 | 0 | 41  (1, 10)  (6, 24) | 101  (4, 21)  (11, 63) | 110  (6, 22)  (13, 65) | 122  (6, 23)  (17, 69) | 123  (6, 23)  (18, 69) |  |  |  |  | 123  (6, 23)  (18, 69) |
| Cumulative SC deaths  (SL, SM)  (NL, NM) | 1953–1979 | 0 | 2  (1, 0)  (0, 1) | 9  (4, 0)  (3, 2) | 22  (17, 0)  (3, 2) | 79  (52, 7)  (14, 5) | 120  (78, 14)  (22, 5) | 173  (112, 19)  (32, 9) | 230  (152, 24)  (42, 11) | 283  (186, 25)  (57, 14) | 335  (214, 29)  (75, 14) | 342  (216, 30)  (78, 15) |
|  | 1980–1989 | 0 | 5  (1, 1)  (1, 2) | 10  (4, 1)  (2, 3) | 18  (8, 3)  (4, 3) | 57  (31, 11)  (7, 7) | 85  (51, 15)  (8, 10) | 115  (62, 19)  (13, 20) | 135  (75, 22)  (14, 22) |  |  | 135  (75, 22)  (14, 22) |
|  | 1990–2015 | 0 | 11  (8, 1)  (0, 1) | 21  (16, 3)  (0, 1) | 46  (30, 5)  (5, 2) | 84  (50, 10)  (12, 5) | 98  (56, 11)  (13, 11) |  |  |  |  | 98  (56, 11)  (13, 11) |
| Cumulative CVD deaths  (SL, SM)  (NL, NM) | 1953–1979 | 0 | 2  (2, 0)  (0, 0) | 14  (14, 0)  (0, 0) | 34  (25, 4)  (4, 0) | 98  (60, 18)  (14, 3) | 134  (84, 24)  (17, 5) | 176  (113, 26)  (26, 7) | 218  (140, 31)  (32, 10) | 259  (164, 39)  (39, 12) | 294  (188, 39)  (49, 12) | 300  (190, 39)  (51, 12) |
|  | 1980–1989 | 0 | 1  (1, 0)  (0, 0) | 10  (5, 2)  (1, 0) | 20  (10, 4)  (3, 1) | 50  (24, 9)  (9, 5) | 65  (32, 12)  (12, 6) | 74  (37, 12)  (13, 9) | 81  (41, 14)  (14, 9) |  |  | 81  (41, 14)  (14, 9) |
|  | 1990–2015 | 0 | 6  (4, 0)  (1, 1) | 7  (10, 2)  (2, 3) | 35  (18, 4)  (3, 6) | 56  (31, 8)  (6, 7) | 59  (32, 8)  (7, 7) |  |  |  |  | 59  (32, 8)  (7, 7) |
| Cumulative OC deaths  (SL, SM)  (NL, NM) | 1953–1979 | 0 | 22  (9, 3)  (4, 6) | 36  (18, 6)  (5, 7) | 49  (24, 8)  (9, 7) | 89  (53, 12)  (16, 7) | 110  (67, 14)  (20, 8) | 134  (85, 15)  (24, 9) | 169  (107, 17)  (31, 12) | 199  (127, 17)  (36, 17) | 247  (158, 19)  (49, 19) | 259  (166, 19)  (53, 19) |
|  | 1980–1989 | 0 | 2  (0, 2)  (0, 0) | 8  (4, 2)  (0, 2) | 18  (10, 2)  (2, 4) | 49  (27, 5)  (9, 8) | 75  (42, 7)  (15, 11) | 94  (53, 9)  (17, 14) | 103  (58, 12)  (18, 14) |  |  | 103  (58, 12)  (18, 14) |
|  | 1990–2015 | 0 | 11  (4, 3)  (3, 0) | 50  (19, 7)  (16, 4) | 81  (33, 9)  (23, 9) | 119  (47, 14)  (30, 14) | 131  (56, 14)  (32, 15) |  |  |  |  | 131  (56, 14)  (32, 15) |
| CI, confidence interval; NL or NM, non-seminoma, localized or metastatic at diagnosis; SL or SM, seminoma, localized or metastatic at diagnosis; TC, testicular cancer; SC, second cancer (excluding TC); CVD, cardiovascular disease; OC, other causes | | | | | | | | | | | | |
