# Supplementary material for: Causes of inferior relative survival after testicular germ cell tumor diagnosed 1953–2015: A population-based prospective cohort study
Source: PLoS One. 2019 Dec 18;14(12):e0225942. doi: 10.1371/journal.pone.0225942 (PMC6919610; doi:10.1371/journal.pone.0225942)
Supplement: S3 Table — (DOCX) [file pone.0225942.s003.docx]

| **S3 Table. Standardized mortality ratios for selected causes of death among testicular germ cell tumor patients diagnosed in Norway.** | | | | | | | | | | |
| --- | --- | --- | --- | --- | --- | --- | --- | --- | --- | --- |
| **Cause of death** | **Cohort of diagnosis** | | | | | | | | | **Code^a^** |
|  | **1953–1979** | | | **1980–1989** | | | **1990–2015** | | |  |
|  | **O** | **SMR (95% CI)** | **SMR by subcategory^b^, O** | **O** | **SMR (95% CI)** | **SMR by subcategory, O** | **O** | **SMR (95% CI)** | **SMR by subcategory, O** |  |
| **Testicular cancer** | 617 |  |  | 76 |  |  | 123 |  |  |  |
| **All non-TC causes** | 901 | **1.42** (1.33–1.52) | A: **1.34** (1.16–1.55), 194  B: **1.42** (1.23–1.64), 200  C: **1.46** (1.34–1.60), 507  D: **1.41** (1.32–1.52), 842 | 319 | **1.36** (1.22–1.52) | A: **1.26** (1.06–1.50), 117  B: **1.38** (1.15–1.67), 120  C: **1.51** (1.22–1.88), 82  D: **1.39** (1.24–1.57), 291 | 288 | **1.21** (1.08–1.35) | A: **1.21** (1.07–1.38), 231  B: 1.18 (0.91–1.54), 57  D: **1.17** (1.02–1.34), 200 | All-TC |
| Seminomas, localized | 572 | **1.34** (1.23–1.46) | A: **1.24** (1.04–1.48),120  B: **1.26** (1.06–1.51), 124  C: **1.42** (1.27–1.59), 328  D: **1.33** (1.22–1.45), 536 | 174 | **1.39** (1.20–1.62) | A: **1.27** (1.01–1.60), 65  B: **1.42** (1.10–1.84), 66  C: **1.58** (1.18–2.15), 43  D: **1.45** (1.23–1.70), 161 | 144 | **1.17** (1.00–1.38) | A: 1.15 (0.97–1.39), 112  B: 1.26 (0.89–1.83), 32  D: 1.13 (0.93–1.38), 99 |  |
| Seminomas, metastatic | 88 | **1.82** (1.43–2.29) | A: **1.75** (1.21–2.58), 26  B: **2.54** (1.67–3.88), 30  C: **1.47** (1.04–2.06), 32  D: **1.86** (1.45–2.38), 82 | 48 | **1.79** (1.36–2.37) | A: **1.58** (1.03–2.49), 19  B: **1.95** (1.20–3.29), 17  C: **1.99** (1.14–3.63), 12  D: **1.86** (1.38–2.50), 43 | 33 | 1.36 (0.98–1.94) | A: **1.48** (1.03–2.19), 29  B: 0.86 (0.34–2.82), 4  D: 1.22 (0.82–1.88), 21 |  |
| Non-seminomas, localized | 182 | **1.49** (1.28–1.73) | A: 1.28 (0.87–1.92), 31  B: **1.53** (1.09–2.19), 36  C: **1.54** (1.28–1.86), 115  D: **1.50** (1.29–1.75), 174 | 46 | 1.05 (0.78–1.42) | A: 1.17 (0.75–1.88), 19  B: 0.97 (0.58–1.70), 16  C: 1.00 (0.57–1.90), 11  D: 1.08 (0.80–1.49), 43 | 52 | 1.06 (0.82–1.38) | A: 1.16 (0.87–1.57), 43  B: 0.75 (0.42–1.46), 9  D: 0.96 (0.70–1.34), 34 |  |
| Non-seminomas, metastatic | 46 | **1.99** (1.38–2.82) | A: **2.51** (1.40–4.69), 14  B: 1.24 (0.59–2.87), 7  C: **2.10** (1.34–3.25), 25  D: **1.73** (1.19–2.48), 37 | 45 | 1.32 (0.98–1.79) | A: 1.05 (0.60–2.00), 12  B: 1.42 (0,89–2.34), 19  C: 1.49 (0,89–2.64), 14  D: 1.27 (0.94–1.76), 40 | 33 | **1.55** (1.04–2.33) | A: 1,41 (0,91–2,28), 22  B: 1,93 (0,92–4,13), 11  D: 1.58 (1.00–2.54), 25 |  |
| **All non-TC second cancers** | 342 | **2.00** (1.79–2.23) | A: **1.70** (1.32–2.22), 55  B: **2.12** (1.71–2.66), 82  C: **2.05** (1.78–2.36), 205  D: **2.03** (1.82–2.27), 333 | 135 | **1.90** (1.61–2.25) | A: **1.57** (1.15–2.20), 38  B: **1.94** (1.50–2.56), 55  C: **2.26** (1.69–3.08), 42  D: **1.90** (1.61–2.27), 125 | 98 | **1.39** (1.15–1.70) | A: **1.27** (1.01–1.62), 69  B: **1.77** (1.24–2.60), 29  D: **1.45** (1.17–1.82), 77 | 2.1-TC |
| *Seminomas, localized* | 216 | **1.89** (1.65–2.16) | A: **1.75** (1.31–2.41), 39  B: **1.71** (1.29–2.30), 46  C: **2.01** (1.68–2.41), 131  D: **1.94** (1.69–2.23), 212 | 75 | **1.95** (1.56–2.45) | A: **1.66** (1.11–2.57), 23  B: **2.01** (1.43–2.90), 31  C: **2.28** (1.51–3.59), 21  D: **2.01** (1.60–2.55), 71 | 56 | **1.46** (1.13–1.91) | A: **1.40** (1.04–1.92), 41  B: **1.65** (1.00–2.89), 15  D: **1.39** (1.03–1.91), 40 |  |
| *Seminomas, metastatic* | 30 | **2.27** (1.61–3.25) | A: 0.57 (0.12–5.50), 2  B: **4.55** (2.68–8.07), 15  C: **2.03** (1.22–3.52), 13  D: **2.44** (1.72–3.51), 30 | 22 | **2.55** (1.72–3.87) | A: 1.74 (0.79–4.50), 6  B: **3.54** (1.96–6.86), 11  C: **2.39** (1.06–6.57), 5  D: **2.71** (1.81–4.18), 21 | 11 | 1.39 (0.78–2.70) | A: 1.44 (0.76–3.04), 9  B: 1.20 (0.26–11.60), 2  D: 1.36 (0.70–3.02), 8 |  |
| *Non-seminomas, localized* | 78 | **2.31** (1.85–2.91) | A: 1.66 (0.83–3.74), 8  B: **2.89** (1.80–4.88), 18  C: **2.29** (1.76–3.02), 52  D: **2.30** (1.83–2.90), 75 | 14 | 1.10 (0.67–1.90) | A: 1.28 (0.53–3.79), 5  B: 0.58 (0.18–2.84), 3  C: 1.64 (0.75–4.22), 6  D: 1.00 (0.59–1.83), 12 | 13 | 1.02 (0.61–1.81) | A: 0.89 (0.47–1.95), 8  B: 1.31 (0.56–3.78), 5  D: 1.31 (0.78–2.35), 13 |  |
| *Non-seminomas, metastatic* | 15 | **2.21** (1.29–3.95) | A: **3.98** (1.59–12.01), 5  B: 1.81 (0.56–8.53), 3  C: 1.81 (0.88–4.22), 7  D: **2.02** (1.15–3.72), 13 | 22 | **2.18** (1.45–3.39) | A: 1.61 (0.59–5.8), 4  B: **2.17** (1.13–4.61), 9  C: **2.61** (1.39–5.46), 9  D: **1.97** (1.28–3.16), 19 | 11 | **2.12** (1.18–4.11) | A: 1.12 (0.41–4.10), 4  B: **4.34** (2.05–10.27), 7  D: **2.43** (1.32–4.88), 10 |  |
| MN, lip, oral cavity, pharynx | 3 | 0.99 (0.31–4.87) | A: 0 (–), 0  B: 2.39 (0.51–23.92), 2  C: 0.64 (–), 1  D: 1.03 (0.32–5.08), 3 | 5 | **3.44** (1.45–10.27) | A: **5.79** (1.81–28.41), 3  B: 3.37 (0.73–33.88), 2  C: 0 (–), 0  D: **2.96** (1.11–10.64), 4  SL: **3.85** (1.20–18.91), 3  NM: **9.07** (1.93–91.14), 2 | 2 | 1.44 (0.31–14.42) | A: 1.86 (0.40–18.72), 2  B: 0 (–), 0  D: **1.89** (0.41–19.03), 2 | 2.1.1 |
| MN, esophagus | 7 | 1.99 (0.97–4.77) | C: **2.74** (1.26–7.21), 4  D: 2.05 (1.00–4.92), 7 | 1 | 0.55 | D: 0.58 (–), 1 | 5 | **2.61** (1.10–7.77) | B: **7.64** (2.88–27.35), 4  D: **3.30** (1.40–9.82), 5  NL: **6.37** (1.40–62.25), 2 | 2.1.2 |
| MN, stomach | 31 | **2.62** (1.86–3.81) | B: **4.29** (2.54–7.85), 13  C: **2.64** (1.56–4.84), 13  D: **2.89** (2.05–4.20), 31  SL: **2.10** (1.33–3.51), 17  NL: **4.15** (2.19–8.82), 9  NM: **8.33** (2.50–41.32), 3 | 13 | **3.98** (2.36–7.26) | A: **4.78** (2.33–11.4), 7  C: **4.65** (1.47–22.68), 3  D: **4.52** (2.68–8.27), 13  SL: **4.42** (2.26–9.84), 8  SM: **9.48** (3.61–32.95), 4 | 5 | 1.90 (0.80–5.66) | B: **5.23** (1.64–25.67), 3  D: **2.58** (1.09–7.71), 5 | 2.1.3 |
| MN, colorectal, anus | 44 | **1.95** (1.46–2.66) | C: **2.25** (1.59–3.27), 31  D: **2.02** (1.51–2.76), 44  SL: **1.92** (1.35–2.83), 29  NL: **2.94** (1.73–5.37), 13 | 11 | 1.17 (0.67–2.24) | C: **2.36** (1.09–6.14), 6  D: 1.26 (0.72–2.42), 11 | 6 | 0.65 (0.30–1.71) | D: 0.85 (0.39–2.23), 6 | 2.1.4 |
| MN, liver, intrahepatic ducts | 11 | **5.68** (3.21–11.07) | B: **8.92** (2.79–43.83), 3  C: **6.22** (3.18–13.93), 8  D: **5.93** (3.35–11.55), 11  SL: **6.30** (3.22–14.10), 8  NL: **4.96** (1.06–49.70), 2 | 1 | 0.91 | D: 0.94 (–), 1 | 2 | 1.34 (0.29–13.48) | D: 0.82 (–), 1 | 2.1.5 |
| MN, pancreas | 28 | **2.97** (2,08–4,41) | B: **4.11** (2.18–8.71), 9  C: **3.50** (2.26–5.71), 19  D: **3.10** (2.16–4.60), 28  SL: **3.04** (1.97–4.95), 19  SM: **5.44** (2.01–19.62), 4 | 18 | **4.31** (2.75–7.13) | B: **6.45** (3.64–12.55), 11  C: **3.96** (1.67–11.83), 5  D: **4.35** (2.74–7.32), 17  SL: **4.01** (2.12–8.49), 9  SM: **7.90** (2.94–28.13), 4  NM: **6.58** (2.48–23.38), 4 | 8 | 1.85 (0.95–4.16) | D: 1.77 (0.81–4.66), 6 | 2.1.6 |
| MN, trachea, bronchus, lung | 47 | 1.25 (0.94–1.69) | A: **2.02** (1.19–3.71), 13  D: 1.26 (0.95–1.71), 46 | 32 | **1.89** (1.35–2.72) | A: **1.82** (1.00–3.67), 10  B: **2.30** (1.43–3.92), 16  D: **1.96** (1.40–2.85), 31  SL: **2.17** (1.42–3.48), 20  SM: **2.80** (1.28–7.23), 6 | 14 | 0.87 (0.53–1.55) | D: 0.97 (0.56–1.82), 12 | 2.1.8 |
| Melanoma | 10 | **2.53** (1.39–5.11) | B: **5.53** (2.34–16.45), 5  D: **2.63** (1.44–5.32), 10  SL: **2.41** (1.10–6.34), 6  SM: **6.41** (1.37–63.71), 2 | 3 | 1.18 (0.37–5.80) | D: 1.27 (0.40–6.25), 3 | 6 | 2.00 (0.91–5.26) | D: 2.21 (0.93–6.61), 5 | 2.1.9 |
| MN, prostate | 39 | **1.46** (1.08–2.04) | A: **2.43** (1.19–5.76), 7  C: **1.53** (1.07–2.25), 29  D: **1.42** (1.04–2.00), 39  SL: **1.47** (1.02–2.20), 27 | 7 | 0.83 (0.40–1.99) | D: 0.63 (0.26–1.88), 5 | 9 | 1.30 (0.69–2.78) | D: 2.25 (0.70–11.08), 3 | 2.1.14 |
| MN, kidney | 13 | **2.64** (1.57–4.83) | C: **3.29** (1.75–6.95), 9  D: **2.76** (1.63–5.04), 13  SM: **5.22** (1.16–50.00), 2  NL: **5.16** (2.16–15.39), 5 | 1 | 0.46 | D: 0.49 (–),1 | 2 | 0.91 (0.20–9.09) | D: 0.59 (–), 1 | 2.1.15 |
| MN, bladder | 18 | **2.71** (1.73–4.48) | A: **4.03** (1.52–14.35), 4  B: **2.96** (1.11–10.63), 4  C: **2.32** (1.28–4.70), 10  D: **2.64** (1.67–4.44), 17  SL: **2.84** (1.67–5.19), 13  NL: **3.30** (1.26–11.56), 4 | 10 | **4.74** (2.60–9.55) | B: **3.44** (1.10–16.43), 3  C: **10.31** (4.66–27.32), 6  D: **5.10** (2.80–10.29), 10  SL: **5.71** (2.80–13.52), 7  NM: **13.83** (4.20–67.81), 3 | 4 | 2.32 (0.87–8.33) | B: **4.73** (1.01–47.50), 2  D: 2.25 (0.70–11. 08), 3 | 2.1.16 |
| MN, brain and CNS | 9 | **2.06** (1.09–4.37) | B: **3.41** (1.29–12.24), 4  D: 1.93 (0.98–4.33), 8  NL: **3.99** (1.49–14.35), 4 | 2 | 0.67 (0.14–6.73) | D: 0.74 (0.16–7.41), 2 | 8 | **1.98** (1.01–4.45) | A: **2.46** (1.26–5.52), 8  D: **2.39** (1.16–5.75), 7  NL: **3.64** (1.93–7.73), 9 | 2.1.17 |
| MN, thyroid | 0 | 0 (–) |  | 0 | 0 (–) |  | 0 | 0 (–) |  | 2.1.18 |
| Hodgkin disease, lymphoma | 6 | 1.32 (0.60–3.47) | D: 1.36 (0.62–3.59), 6  SM: **5.66** (1.20–56.57), 2 | 1 | 0.44 | D: 0.46 (–), 1 | 2 | 0.97 (0.21–9.71) | D: 0.66 (–), 1 | 2.1.19 |
| Leukemia | 8 | 1.60 (0.82–3.60) | D: 1.71 (0.87–3.85), 7 | 3 | 1.59 (0.50–7.84) | D: 1.76 (0.55–8.65), 3 | 6 | **3.47** (1.59–9.15) | A: **3.83** (1.62–11.39), 5  D: **3.04** (1.14–10.90), 4  SL: **4.21** (1.59–15.03), 4 | 2.1.20 |
| MN, other lymph./hematol.^c^ | 9 | **1.89** (1.00–4.00) | A: **3.91** (1.47–14.03), 4  D: **1.76** (0.90–3.95), 8  SL: **2.16** (1.05–5.19), 7 | 2 | 1.65 (0.26–12.1) | D: 0.68 (–), 1 | 1 | 0.71 | D: 0 (–), 0 | 2.1.21 |
| MN, other (no TC deaths) | 59 | **3.50** (2.73–4.57) | A: **2.31** (1.23–4.89), 9  B: **3.08** (1.78–5.81), 12  C: **4.20** (3.07–5.89), 38  D: **3.55** (2.75–4.67), 56  SL: **2.58** (1.81–3.81), 29  SM: **6.90** (3.73–14.14), 9  NL: **5.71** (3.68–9.30), 19 | 24 | **3.68** (2.50–5.65) | A: **2.35** (1.07–6.23), 6  B: **4.39** (2.47–8.57), 11  C: **4.78** (2.32–11.50), 7  D: **3.37** (2.21–5.40), 20  SL: **3.13** (1.76–6.12), 11  SM: **6.30** (2.67–18.36), 5  NM: **5.34** (2.27–15.66), 5 | 17 | **2.72** (1.72–4.57) | A: **2.43** (1.41–4.56), 12  B: **3.83** (1.61–11.45), 5  D: **2.63** (1.53–4.95), 12  SL: **2.98** (1.63–6.04), 10 | 2.1.22 |
| **Cardiovascular disease** | 300 | **1.12** (1.00–1.26) | A: 1.03 (0.81–1.32), 68  B: 1.15 (0.92–1.46), 75  C: 1.15 (0.99–1.35), 157  D: **1.14** (1.01–1.28), 286 | 81 | 1.07 (0.85–1.34) | A: 1.26 (0.94–1.72), 43  B: 0.93 (0.63–1.41), 25  C: 0.86 (0.51–1.58), 13  D: 1.07 (0.84–1.37), 71 | 59 | 0.96 (0.75–1.26) | A: 1.08 (0.83–1.44), 53  B: 0.48 (0.22–1.27), 6  D: 0.94 (0.70–1.30), 42 | 7. |
| *Seminomas, localized* | 190 | 1.03 (0.90–1.19) | A: 0.93 (0.69–1.28), 42  B: 1.04 (0.79–1.40), 48  C: 1.08 (0.89–1.32), 100  D: 1.02 (0.88–1.18), 172 | 41 | 0.96 (0.70–1.32) | A: 1.04 (0.70–1.63), 21  B: 0.87 (0.52–1.57), 13  C: 0.90 (0.43–2.18), 7  D: 0.97 (0.70–1.38), 36 | 32 | 0.98 (0.70–1.41) | A: 1.11 (0.78–1.62), 29  B: 0.46 (0.14–2.25), 3  D: 0.94 (0.62–1.47), 22 |  |
| *Seminomas, metastatic* | 39 | **1.88** (1.33–2.66) | A: **1.90** (1.17–3.27), 13  B: **2.22** (1.19–4.36), 12  C: 1.65 (0.96–2.91), 14  D: **2.08** (1.45–2.99), 39 | 14 | 1.51 (0.89–2.70) | A: 1.86 (0.98–3.89), 9  B: 1.10 (0.34–5.26), 3  C: 1.18 (0.26–10.71), 2  D: 1.56 (0.88–2.94), 12 | 8 | 1.25 (0.65–2.67) | A: 1.34 (0.68–3.04), 7  B: 0.83 (–), 1  D: 1.31 (0.62–3.27), 6 |  |
| *Non-seminomas, localized* | 51 | 1.05 (0.80–1.39) | A: 0.98 (0.50–2.07), 10  B: 0.88 (0.46–1.85), 9  C: 1.14 (0.81–1.63), 32  D: 1.10 (0.84–1.46), 51 | 14 | 1.07 (0.62–1.95) | A: 1.72 (0.86–3.71), 9  B: 0.62 (0.18–3.10), 3  C: 0.66 (0.16–5.78), 2  D: 1.10 (0.62–2.06), 13 | 7 | 0.59 (0.28–1.47) | A: 0.69 (0.30–1.87), 6  B: 0.32 (–), 1  D: 0.57 (0.23–1.74), 5 |  |
| *Non-seminomas, metastatic* | 12 | 1.42 (0.75–2.79) | A: 0.94 (0.20–9.01), 2  B: 1.33 (0.43–5.98), 3  C: 1.72 (0.71–4.49), 7  D: 1.53 (0.80–3.02), 12 | 9 | 1 (0.52–2.14) | A: 0.68 (0.14–6.79), 2  B: 1.34 (0.54–4.09), 5  C: 0.86 (0.18–8.60), 2  D: 1.07 (0.55–2.30), 9 | 7 | 1.50 (0.64–3.92) | A: **2.23** (1.00–5.61), 7  B: 0 (–), 0  D: 1.07 (0.35–4.14), 4 |  |
| Ischemic heart diseases | 169 | 1.06 (0.91–1.24) | D: 1.08 (0.92–1.26), 160  SM: **1.74** (1.13–2.73), 22 | 52 | 1.21 (0.92–1.60) | D: 1.20 (0.90–1.63), 44 | 30 | 0.92 (0.65–1.34) | D: 0.94 (0.63–1.48), 22 | 7.1 |
| *Acute myocardial infarction* | 119 | 1.06 (0.89–1.28) | D: 1.10 (0.92–1.33), 114  SM: **2.01** (1.25–3.33), 18 | 39 | 1.34 (0.99–1.87) | A: **1.51** (1.02–2.34), 23  D: 1.26 (0.90–1.84), 31  SL: **1.48** (1.01–2.27), 24 | 18 | 0.84 (0.54–1.40) | D: 0.73 (0.41–1.43), 11 | 7.1.1 |
| Non-ischemic heart diseases | 50 | **1.59** (1.20–2.13) | B: **1.86** (1.02–3.77), 10  C: **1.55** (1.12–2.21), 35  D: **1.59** (1.21–2.14), 49  SL: **1.43** (1.01–2.09), 31  SM: **2.59** (1.12–6.99), 6  NM: **4.63** (1.69–14.80), 5 | 16 | 1.42 (0.87–2.46) | D: 1.43 (0.86–2.53), 15  SM: **4.96** (2.25–12.86), 6 | 13 | 1.25 (0.74–2.31) | D: 1.02 (0.52–2.31), 8 | 7.2 |
| Cerebrovascular diseases | 44 | 0.88 (0.66–1.20) | D: 0.90 (0.67–1.23), 42 | 5 | 0.37 (0.16–1.09) | D: 0.42 (0.18–1.23), 5 | 13 | 1.20 (0.71–2.21) | D: 1.13 (0.60–2.41), 9 | 7.3 |
| Other circulatory diseases | 37 | **1.39** (1.02–1.96) | D: **1.40** (1.01–1.99), 35  SM: **3.94** (1.96–8.87), 8 | 8 | 1.02 (0.51–2.30) | D: 0.99 (0.48–2.39), 7 | 3 | 0.45 (0.14–2.17) | D: 0.59 (0.19–2.90), 3 | 7.4 |
| **Other or unknown causes** | 259 | **1.34** (1.18–1.51) | A: **1.53** (1.22–1.95), 71  B: 1.15 (0.86–1.57), 43  C: **1.32** (1.13–1.55), 145  D: **1.24** (1.09–1.41), 223 | 103 | 1.19 (0.98–1.45) | A: 1.04 (0.77–1.46), 36  B: 1.27 (0.93–1.77), 40  C: 1.31 (0.90–1.95), 27  D: **1.24** (1.02–1.52), 95 | 131 | **1.23** (1.04–1.46) | A: **1.25** (1.04–1.51), 109  B: 1.12 (0.75–1.75), 22  D: 1.11 (0.90–1.38), 81 | All-2.1-7-TC |
| *Seminomas, localized* | 166 | **1.30** (1.12–1.52) | A: 1.34 (0.98–1.86), 39  B: 1.20 (0.84–1.75), 30  C: **1.33** (1.10–1.61), 97  D: **1.24** (1.06–1.45), 148 | 58 | **1.33** (1.03–1.73) | A: 1.22 (0.82–1.89), 21  B: 1.37 (0.89–2.18), 22  C: 1.47 (0.91–2.50), 15  D: **1.40** (1.07–1.84), 54 | 56 | 1.09 (0.85–1.43) | A: 1.01 (0.76–1.38), 42  B: 1.42 (0.85–2.54), 14  D: 1.05 (0.77–1.47), 37 |  |
| *Seminomas, metastatic* | 19 | 1.31 (0.82–2.18) | A: **2.44** (1.33–4.86), 11  B: 0.97 (0.31–4.45), 3  C: 0.73 (0.30–2.10), 5  D: 1.01 (0.58–1.85), 13 | 12 | 1.35 (0.77–2.51) | A: 1.06 (0.41–3.63), 4  B: 1.04 (0.32–5.06), 3  C: 2.23 (0.87–6.82), 5  D: 1.29 (0.71–2.57), 10 | 14 | 1.42 (0.85–2.55) | A: 1.61 (0.93–3.01), 13  B: 0.56 (–), 1  D: 1.03 (0.50–2.44), 7 |  |
| *Non-seminomas, localized* | 53 | **1.33** (1.02–1.76) | A: 1.42 (0.84–2.59), 13  B: 1.28 (0.68–2.70), 9  C: 1.31 (0.92–1.90), 31  D: 1.30 (0.98–1.74), 48 | 18 | 1.01 (0.64–1.67) | A: 0,70 (0,30–2,06), 5  B: 1.54 (0.83–3.14), 10  C: 0.70 (0.21–3.49), 3  D: 1.13 (0.71–1.89), 18 | 32 | 1.30 (0.94–1.85) | A: 1.49 (1.06–2.17), 29  B: 0.58 (0.20–2.61), 3  D: 0.96 (0.61–1.60), 16 |  |
| *Non-seminomas, metastatic* | 19 | **2.39** (1.51–3.89) | A: **3.20** (1.49–7.89), 7  B: 0.57 (–), 1  C: **2.75** (1.54–5.10), 11  D: **1.69** (1.00–2.98), 12 | 14 | 0.93 (0.56–1.65) | A: 1.01 (0.46–2.65), 6  B: 0.91 (0.39–2.66), 5  C: 0.82 (0.25–4.06), 3  D: 0.90 (0.52–1.69), 12 | 15 | 1.32 (0.78–2.34) | A: 1.25 (0.70–2.44), 11  B: 1.56 (0.51–6.01), 4  D: 1.38 (0.75–2.76), 11 |  |
| Infectious / parasitic diseases | 8 | 1.12 (0.57– 2.52) | D: 0.89 (0.41– 2.35), 6 | 6 | 1.82 (0.83–4.79) | D: 1.62 (0.68–4.83), 5  SM: **5.84** (1.23–58.57), 2 | 10 | **3.06** (1.69–6.18) | A: **3.64** (1.94–7.68), 9  D: **2.47** (1.14–6.44), 6  NL: **4.84** (1.50–23.71), 3 | 1. |
| *Viral hepatitis* | 0 | 0 (–) | D: 0 (–), 0 | 1 | 22.86 (–) | D: 22.86 (–), 1 | 3 | **32.49** (10.19–159.5) | A: **43.56** (13.67–213.7), 3  D: **26.69** (5.76–267.7), 2  SL: **40.03** (8.64–400.9), 2 | 1.3 |
| *Other infectious diseases*^d^ | 7 | 1.32 (0.64–3.18) | D: 0.97 (0.41–2.89), 5 | 3 | 1.40 (0.44–6.84) | D: 1.43 (0.45–7.02), 3 | 5 | **2.38** (1.00–7.10) | A: **2.74** (1.03–9.87), 4  D: 1.75 (0.55–8.56), 3 | 1.4 |
| Non-MN, benign / uncertain | 8 | **2.62** (1.34–5.88) | C: **3.06** (1.40–8.05), 6  D: **2.74** (1.40–6.15), 8 | 2 | 2.28 (0.49–22.85) | D: 2.43 (0.52–24.37), 2 | 1 | 1.39 (–0) | D: 1.79 (–), 1 | 2.2 |
| Endocrine, nutr., metab.^e^ | 15 | 1.50 (0.92–2.62) | B: **2.66** (1.12–7.93), 5  D: 1.57 (0.96–2.73), 15 | 10 | **2.06** (1.13–4.19) | D: **1.99** (1.05–4.22), 9  SL: **2.37** (1.08–6.23), 6  SM: **5.63** (1.74–27.48), 3 | 3 | 0.54 (0.17–2.64) | D: 0.49 (0.11–4.88), 2 | 4. |
| *Diabetes mellitus* | 12 | 1.51 (0.88–2.84) | D: 1.58 (0.91–2.96), 12 | 6 | 1.63 (0.75–4.30) | D: 1.75 (0.80–4.61), 6  SM: **4.83** (1.04–47.53), 2 | 1 | 0.25 (–) | D: 0.33 (–), 1 | 4.1 |
| *Other* | 3 | 1.73 (0.54–8.50) | B: **6.79** (1.46–68.13)  D: 1.77 (0.55–8.70), 3 | 4 | **4.17** (1.57–15.01) | A: **8.38** (1.80–84.23), 2  B: **4.74** (1.02–47.50), 2  D: **3.23** (1.01–15.88), 3 | 2 | 1.88 (0.40–18.90) | D: 1.23 (–), 1 | 4.2 |
| Mental, behavioral disorders | 6 | 0.49 (0.23–1.28), 6 | D: 0.50 (0.23–1.31), 6 | 6 | 0.75 (0.34–1.97) | D: 0.69 (0.29–2.04), 5 | 11 | 1.21 (0.68–2.36) | D: 0.86 (0.36–2.57), 5 | 5. |
| Nervous system, sense organs | 15 | 1.07 (0.66–1.87) | D: 1.12 (0.69–1.95), 15 | 3 | 0.45 (0.14–2.22) | D: 0.16 (–), 1 | 13 | **1.72** (1.02–3.14) | A: **2.08** (1.21–3.90), 12  D: 1.58 (0.93–8.86), 9  SL: **2.02** (1.04–4.51), 8 | 6. |
| *Alzheimer’s disease* | 2 | 0.73 (0.16–7.26) | D: 0.73 (0.16–7.26), 2 | 0 | 0 (–) | D: 0 (–), 0 | 4 | **3.85** (1.46–13.57) | A: **5.68** (2.18–19.81), 4  D: **4.64** (1.76–16.36), 4  SL: **5.43** (1.74–25.76), 3 | 6.2 |
| Respiratory system diseases | 44 | 0.88 (0.66–1.20) | D: 0.88 (0.65–1.20), 42 | 14 | 0.89 (0.55–1.56) | D: 0.97 (0.59–1.69), 14 | 10 | 0.74 (0.41–1.50) | D: 0.38 (0.14–1.37), 4 | 8. |
| *Pneumonia* | 18 | 0.93 (0.59–1.53) | D: 0.98 (0.62–1.62), 18 | 5 | 1.08 (0.47–3.04) | D: 1.22 (0.54–3.45), 5 | 4 | 1.20 (0.44–4.34) | D: 0.80 (0.17–8.08), 2 | 8.2 |
| *Chronic lower respiratory* | 23 | 0.91 (0.61–1.41) | D: 0.89 (0.59–1.40), 22 | 4 | 0.44 (0.17–1.57) | D: 0.47 (0.18–1.67), 4 | 4 | 0.50 (0.19–1,79) | D: 0.16 (–), 1 | 8.3 |
| *Other respiratory diseases* ^f^ | 3 | 0.77 (0.24–3.79) | A: **4.87** (1.06–48.11), 2  D: 0.53 (0.11–5.29), 2 | 5 | **3.35** (1.41–10.00) | A: **6.80** (1.46–67.74), 2  B: **4.63** (1.45–22.63), 3  D: **3.48** (1.46–10.4), 5  SL: **3.50** (1.08–17.23), 3  NM: **11.90** (2.65–112.0), 3 | 1 | 0.71 (–) | D: 0 (–), 0 | 8.4 |
| Digestive system diseases | 50 | **2.83** (2.16–3.78) | B: **3.20** (1.89–5.87), 13  C: **3.26** (2.31–4.73), 31  D: **2.88** (2.19–3.88), 48  SL: **2.89** (2.09–4.12), 34  NL: **2.86** (1.57–5.75), 10  NM: **5.64** (2.05–20.39), 4 | 19 | **2.51** (1.62–4.11) | B: **3.44** (1.88–7.00), 10  D: **2.64** (1.68–4.38), 18  SL: **3.01** (1.73–5.68), 12  NL: **2.78** (1.03–10.00), 4 | 9 | 1.21 (0.64–2.57) | D: 1.26 (0.61–3.04), 7  SL: **1.98** (1.01–4.45), 8 | 9. |
| *Ulcers, stomach–jejunum* | 11 | **3.78** (2.13–7.38) | B: **6.09** (2.29–21.84), 4  C: **3.23** (1.36–9.64), 5  D: **3.31** (1.75–7.03), 9  SL: **3.45** (1.67–8.31), 7  NL: **7.80** (2.89–28.14), 4 | 1 | 1.21 | D: 1.44 (–), 1 | 2 | 3.32 (0.71–33.43) | D: 4.41 (0.94–44.31), 2  SL: **6.06** (1.30–60.91), 2 | 9.1 |
| *Cirrhosis, fibrosis, c. hep.*^g^ | 5 | 1.02 (0.43–3.03) | D: 1.07 (0.45–3.21), 5 | 8 | **2.57** (1.31–5.77) | A: **2.83** (1.06–10.13), 4  D: **2.91** (1.49–6.53), 8  NL: **6.40** (2.41–22.78), 4 | 3 | 0.96 (0.30–4.74) | D: 0.88 (0.19–8.87), 2 | 9.2 |
| *Other digestive diseases* | 34 | **3.64** (2.62–5.20) | B: **3.95** (1.91–9.51), 7  C: **4.20** (2.86–6.40), 25  D: **3.82** (2.74–5.45), 34  SL: **3.60** (2.42–5.59), 23  NL: **3.42** (1.56–8.92), 6  NM: **12.56** (4.30–47.14), 4 | 10 | **2.98** (1.62–6.05) | B: **4.40** (1.99–11.68), 6  D: **2.89** (1.53–6.15), 9  SL: **3.73** (1.80–8.98), 7 | 4 | 1.24 (0.47–4.47) | D: 1.20 (0.38–5.92), 3 | 9.3 |
| Skin, subcutaneous diseases | 1 | 2.93 (–) | D: 2.93 (–), 1 | 0 | 0 (–) | D: 0 (–), 0 | 0 | 0 (–) | D: 0 (–), 0 | 10. |
| Musculoskeletal, connective^h^ | 1 | 0.49 (–) | D: 0.51 (–), 1 | 0 | 0 (–) | D: 0 (–), 0 | 0 | 0 (–) | D: 0 (–), 0 | 11. |
| Genitourinary diseases | 21 | **2.31** (1.54–3.64) | C: **2.70** (1.69–4.58), 16  D: **2.50** (1.66–3.94), 21  SL: **2.55** (1.61–4.29), 16 | 2 | 0.87 (0.19–8.69) | D: 0.93 (0.20–9.37), 2 | 7 | **3.76** (1.82–8.99) | A: **5.18** (2.52–12.33), 7  D: **2.70** (1.01–9.68), 4  SL: **4.20** (1.59–14.73), 4  NL: **5.61** (1.19–53.83), 2 | 12. |
| *Kidney and ureter* | 18 | **3.04** (1.96–5.00) | C: **3.64** (2.20–6.46), 14  D: **3.29** (2.11–5.40), 18  SL: **3.16** (1.89–5.71), 13 | 1 | 0.65 (–) | D: 0.69 (–), 1 | 4 | **3.18** (1.20–11.30) | A: **4.37** (1.65–15.46), 4  D: **2.99** (0.94–14.61), 3  SL: **4.60** (1.47–21.89), 3 | 12.1 |
| *Other* | 3 | 1.01 (0.32–4.89) | D: 1.09 (0.34–5.28), 3 | 1 | 1.44 (–) | D: 1.59 (–), 1 | 3 | **5.68** (1.76–27.80) | A: **7.96** (2.47–38.86), 3  D: 2.37 (–), 1  NL: **18.50** (3.87–175.0), 2 | 12.2 |
| Congenital, chromosomal | 3 | **4.94** (1.55–24.27) | D: **6.24** (1.96–30.61), 3  SL: **6.30** (1.36–62.99), 2 | 0 | 0 (–) | D: 0 (–), 0 | 1 | 1.62 (–) | D: 0 (–), 0 | 15. |
| Symptoms, signs, ill-defined | 43 | **1.89** (1.41–2.58) | A: **3.64** (2.52–5.44), 27  D: 0.97 (0.64–1.55), 20  SM: **3.99** (1.87–9.84), 7  NM: **10.43** (4.93–24.43), 8 | 7 | 0.97 (0.47–2.32) | D: 1.10 (0.53–2.64), 7 | 6 | 0.85 (0.39–2.24) | D: 1.17 (0.54–3.08), 6 | 16. |
| External causes | 36 | 0.94 (0.68–1.33) | D: 0.92 (0.65–1.34), 30 | 27 | 1.14 (0.79–1.70) | D: 1.27 (0.86–1.95), 24 | 58 | **1.47** (1.15–1.93) | A: **1.33** (1.01–1.81), 46  B: **2.48** (1.43–4.66), 12  D: **1.49** (1.08–2.10), 36  NM**: 2.42** (1.43–4.42), 13 | 17. |
| *Accidents* | 25 | 0.90 (0.62–1.38) | D: 0.89 (0.59–1.41), 21 | 17 | 1.15 (0.73–1.93) | D: 1.25 (0.77–2.18), 15 | 35 | **1.46** (1.06–2.07) | B: **2.59** (1.33–5.79), 8  D: **1.53** (1.03–2.38), 23  NM: **2.18** (1.06–5.24), 7 | 17.1 |
| *Suicide* | 10 | 1.02 (0.56–2.08) | D: 1.02 (0.56–2.08), 8 | 10 | 1.23 (0.68–2.49) | D: 1.23 (0.68–2.49), 9 | 22 | **1.54** (1.03–2.42) | D: 1.54 (0.90–2.78), 13  NM: **2.99** (1.37–7.86), 6 | 17.2 |
| Causes of death are classified according to S1 Table. Statistically significant results (P = <0.05) are highlighted in **bold**.  CI, confidence interval; O, observed deaths in the study population; MN, malignant neoplasm; SMR, standardized mortality ratio; TC, testicular cancer.  ^a^ Code for cause of death as defined in S1 Table.  ^b^ Selected subgroups with SMRs pertaining to follow-up time, histology and disease extent at TGCT diagnosis, given in parentheses with 95% CI: A, <16 years follow-up only; B, 16-<26 years follow-up only; C, ≥26 years follow-up only; D, ≥5 years follow-up only; SL: seminoma, localized; SM: seminoma, metastatic; NL: nonseminoma, localized; NM: nonseminoma, metastatic.  ^c^ Other malignant neoplasms of lymphoid and hematopoietic tissue.  ^d^ Not including tuberculosis (group 1.1) and acquired immunodeficiency syndrome (AIDS, code 1.2).  ^e^ Endocrine, nutritional and metabolic diseases.  ^f^ Not including influenza (group 8.1).  ^g^ Chronic hepatitis.  ^h^ Musculoskeletal and connective tissue diseases. | | | | | | | | | | |
