# Supplementary material for: Causes of inferior relative survival after testicular germ cell tumor diagnosed 1953–2015: A population-based prospective cohort study
Source: PLoS One. 2019 Dec 18;14(12):e0225942. doi: 10.1371/journal.pone.0225942 (PMC6919610; doi:10.1371/journal.pone.0225942)
Supplement: S4 Table — (DOCX) [file pone.0225942.s004.docx]

| **S4 Table. Summary of selected publications reporting long-term cause-specific mortality data among testicular cancer patients.** | | | | | |
| --- | --- | --- | --- | --- | --- |
| **Publication** | **# of patients, histology and stage at diagnosis** | **Time and location of diagnosis** | **Follow-up time** | **Initial treatment (# of patients)** | **Selected standardized mortality ratios (95 % CI), and/or other selected findings** |
| Hanks et al, 1992 [1] | 387, seminoma, stage I-II | 1973 to 1974, US | 1990 (mail survey) | - Infradiaphragmatic RT (387)  - Supradiaphragmatic RT (161) | - SC: 3.4 (no CI, P = <0.001)  - Cardiac death: 2.3 (no CI, P = <0.001)  - Non-cancer death: 3.1 (no CI, P = <0.01)  - Other findings: eight of 10 patients dead of cardiac disease had received supradiaphragmatic RT |
| Horwich 1994 [2] | 859, seminoma, stage I | 1961 to 1985, UK | End of 1989. 8459 person years. | Infradiaphragmatic RT (859) | - SC, overall: 0.90 (0.58 to 1.40). Bladder cancer: 3.80 (1.33 to 10.87). Leukemia: 5.45 (1.99 to 14.9) |
| Fosså 2004 [3] | 3378, malignant germ cell tumor, any stage. Age <56 years at diagnosis, censored at 60 years | 1962 to 1997, CRN data, Norway | End of 1997. 41 960 person years. | Any, but no individual treatment data | - SC, overall: 2.0 (1.7 to 2.4). Stomach cancer 3.0 (1.6 to 5.0). Pancreatic cancer 2.9 (1.? to 5.4). Lung cancer 1.7 (1.1 to 2.6)  - CVD: overall 1.2 (1.0 to 1.5), myocardial infarction 1.1 (0.9 to 1.4)  - OC: GI disorders 2.1 (1.1 to 3.5). Accidents, poisoning, suicide 0.8 (0.6 to 1.2) |
| Zagars 2004 [4] | 453, seminoma, stage I-II | 1951 to 99, MD Anderson Cancer Center, Texas | Median 13.3 years, range = 1.4 to 42.8 years | - Abdominal RT (453)  - PMI (71) | - All causes: 1.59 (1.30 to 1.93). SC: 1.91 (1.14 to 2.98). CVD: 1.61 (1.21 to 2.24). Significant beyond 15 years of follow-up only  - CVD: overall 1.61 (1.21 to 2.24). Findings were significant beyond 15 years of follow-up only  - Other findings: Actuarial survival rates at 10-20-30-40 years were 93-79-59-26%. PMI the only factor correlated with survival in univariate analysis |
| Hem 2004 [5] | Unknown, any histology and stage | 1960 to 99, CRN data, Norway | 59 858 person years (TC patients) | Any, but no individual treatment data | - Suicide: 1.08 (0.62 to 1.76) among TC patients  - Several cancer forms were included in the study |
| Fosså 2007 [6] | 38 907, TGCT, any stage. Only survivors of at least one year | 1943 to 2002, 14 registries in US/Europe including Norway  (1953 to 1999) | Median 10 years, range = 1 to 55 years | - Surgery (8802)  - RT (12454)  - CT (4586)  - RT + CT (777)  - Other / unspecified (459) | - Non-cancer, overall: 1.06 (1.02 to 1.10)  - CVD, overall: 0.98 (0.94 to 1.04), of which hypertensive disorders 1.39 (1.01 to 1.89). CVD by initial treatment >1975: Surgery 0.91 (0.76 to 1.10), CT 1.44 (1.06 to 1.91), RT 0.95 (0.84 to 1.08), CT+RT 2.06 (1.27 to 3.14).  - OC: Infections 1.28 (1.12 to 1.47), of which intestinal 9.10 (4.73 to 16.00). Digestive diseases 1.44 (1.26 to 1.64). Respiratory diseases 1.15 (0.99 to 1.34). Genitourinary diseases 1.27 (0.96 to 1.6). Endocrine/metabolic disorders 1.17 (0.95 to 1.44). Suicide 0.99 (0.83 to 1.18) |
| Schairer 2007 [7] | 29 356, any histology except spermatocytic tumors, any stage. | 1973 to 2002 (SEER, US) | End of 2002 | Any. Initial treatment data only for the 621 patients who developed a SC | - Reporting hazard rate ratios of second cancers in TC patients relative to first cancers for a matched sample. All-cause mortality not significantly increased.  - Among patients diagnosed during 1973 to 1979, increased cancer-specific and all-cause mortality for second cancers in the RT field |
| Robinson 2007 [8] | 9892 (5555 seminoma, 3733 non-seminoma), any stage | 1960 to 2004 (UK) | End of 2004. 104 622 person-years | Any, but no individual treatment data | - Overall SMRs decreasing with later decade of diagnosis, from 8.62 to 1.69 in seminomas and from 56.76 to 8.50 in non-seminomas.  - Relative survival data with up to 20 years of follow-up |
| Powles 2008 [9] | 199, seminoma, stage I | 1986 to 2007 (UK) | Median 9 years, 1841 person years | Carboplatin (199) | -All-cause mortality 0.89 (0.36 to 1.83)  -CVD 1.44 (0.39 to 3.69) |
| Alanee 2012 [10] | 23 381, any histology and stage | 1995 to 2008, SEER, US | End of 2008. 126 762 person years | Any. RT (8153) | -Suicide, overall 1.21 (1.08 to 2.14). Patients aged <30 years at diagnosis: 1.53 (1.09 to 2.09) |
| Beard 2013 [11] | 9193, seminoma, stage I. Age 15 to 70 years at diagnosis | 1973 to 2001, SEER, US | Median 12.7 years if RT, 10.9 years if not. 121 037 person years | - RT (7179)  - Surgery (2014) | - SC: overall 1.81 (1.61 to 2.03). If RT: 1.89 (1.67 to 2.14). Pancreatic cancer 2.54 (1.12 to 5.92) if no initial RT, 3.35 (2.32 to 4.84) if initial RT  - CVD: overall 0.91 (0.80 to 1.05). If RT: 0.89 (0.76 to 1.04)  - OC: suicide 1.45 (1.06 to 1.98), infection 2.32 (1.80 to 3.00), COPD 0.93 (0.63 to 1.38) |
| Horwich 2014 [12] | 2543, seminoma, stage I | 1960 to 1992, 12 cancer centers (11 UK, 1 Norway) | Median 21.8 years, 51 151 person years | - Abdominal RT (2543).  - Mediastinum / neck-RT (25) | - Overall: 1.06 (0.98 to 1.14)  - SC, non-TC: 1.46 (1.30 to 1.65), of which abdominopelvic 1.62 (1.43 to 1.83)  - CVD: overall 0.80 (0.70 to 0.92)  - OC: overall 0.85 (0.72 to 1.00) |
| Gandaglia 2014 [13] | 31 330. Between 1990 and 2009: 16151 seminoma and 11324 nonseminoma, any stage | 1973 to 2009, SEER, US | Median 92.0 months | Any.  - Orchiectomy (19480)  - RT (11850) | - SMRs not reported  - At 15 years of follow-up, cancer-specific mortality was 1.2%, other-cancer mortality was 1.1%, non-cancer mortality was 2.9%. Mortality was higher in patients with distant disease at diagnosis, as well as higher age. |
| Fung 2015 [14] | 15006, non-seminoma, any stage | 1980 to 2010, SEER, US | Median 7.9 years / 81 227 person years (surgery), 6.5 years / 60 065 person years (CT) | Either initial CT (6909) or initial surgery alone (8097) without RT. | -Non-cancer, surgery only 0.96 (0.84 to 1.11), CT only 1.60 (1.40 to 1.82)  -CVD, surgery only 0.81 (0.60 to 1.07).  -CVD, CT only 1.36 (1.03 to 1.78), of which patients diagnosed 2000 to 2010 1.97 (1.13 to 3.20). Cerebrovascular diseases 2.40 (1.15 to 4.42)  -CVD during first year of diagnosis: 5.31 (2.65 to 9.51)  -OC after initial CT: septicemia 7.14 (3.41 to 13.14). Pneumonia / influenza 3.05 (1.12 to 6.65). Symptoms/signs/ill-defined conditions 4.31 (2.36 to 7.23)  -OC regardless of initial treatment: increased SMRs from benign neoplasms and those of unknown behavior  -Other findings: increased CVD mortality with extent of disease and increasing age at TC diagnosis |
| Kier 2016 [15] | 2804, seminoma, any stage. 2386, non-seminoma, any stage. | 1984-2007, Denmark | Median 14.4 years, until December 31^st^, 2012 | - Surveillance (2985)  - BEP (1432)  - RT (588)  - MTOL (86) | Risk for death from all causes, including GCC, was increased 2 times after BEP, 1.3 times after RT, 16 times after MTOL and reduced after surveillance (HR, 0.9). Second malignant neoplasms caused excess mortality by 1.6 times after BEP, by 2.1 times after RT, and by 5.8 times after MTOL |
| Gunnes 2017 [16] | 738, any histology and stage. Other cancer patients also included. | Born during 1965 to 1985. Diagnosed with TC before age 25, Norway | Through 2008 | Any, but no individual treatment data | - A hazard ratio for suicide of 2.9, 95% CI = 1.3 to 6.4 was found for TC survivors.  - Other cancers with a particularly elevated suicide risk was CNS tumors, leukemia and bone/soft tissue sarcomas. |
| Patel 2017 [17] | 16 463, seminoma, stage I-II | 1998-2013, SEER, US | Median 99.5 months | - No RT (7337)  - RT (9 126) | RT associated with reduced overall- and cancer-specific survival among stage IA patients due to an almost twofold risk of SC |
| Zhang 2019 [18] | 4879, seminoma, any stage. 3717, nonseminoma, any stage. | 1980-2015, Swedish Cancer Registry data | Median 11 years | Any | - SMRs not reported  - Survival at 30 years of follow-up was approximately 80% for TC patients without SC and 40% for patients with SC. |
| BEP, bleomycin, etoposide, cisplatin; CNS, central nervous system; COPD, chronic obstructive pulmonary disease; CRN, Cancer Registry of Norway; CVD, cardiovascular disease; CT, chemotherapy; GCC, germ cell cancer; MTOL, more than one line of treatment; OC, other causes of death (not SC or CVD); PMI, prophylactic mediastinal irradiation; RT, radiotherapy; SC, second cancer; SEER, Surveillance, Epidemiology, and End Results; SMR, standardized mortality ratio; TC, testicular cancer; TGCT, testicular germ cell tumor; UK, United Kingdom; US, United States. | | | | | |

**References, S4 Table**

1. Hanks GE, Peters T, Owen J. Seminoma of the testis: long-term beneficial and deleterious results of radiation. International journal of radiation oncology, biology, physics. 1992;24:913-9. PubMed PMID: 1447034.

2. Horwich A, Bell J. Mortality and cancer incidence following radiotherapy for seminoma of the testis. Radiotherapy and oncology : journal of the European Society for Therapeutic Radiology and Oncology. 1994;30:193-8. PubMed PMID: 8209001.

3. Fosså SD, Aass N, Harvei S, Tretli S. Increased mortality rates in young and middle-aged patients with malignant germ cell tumours. British journal of cancer. 2004;90:607-12. doi: 10.1038/sj.bjc.6601558. PubMed PMID: 14760372.

4. Zagars GK, Ballo MT, Lee AK, Strom SS. Mortality after cure of testicular seminoma. Journal of clinical oncology : official journal of the American Society of Clinical Oncology. 2004;22:640-7. doi: 10.1200/JCO.2004.05.205. PubMed PMID: 14726503.

5. Hem E, Loge JH, Haldorsen T, Ekeberg O. Suicide risk in cancer patients from 1960 to 1999. J Clin Oncol. 2004;22(20):4209-16. Epub 2004/10/16. doi: 10.1200/JCO.2004.02.052. PubMed PMID: 15483032.

6. Fosså SD, Gilbert E, Dores GM, Chen J, McGlynn KA, Schonfeld S, et al. Noncancer causes of death in survivors of testicular cancer. Journal of the National Cancer Institute. 2007;99:533-44. doi: 10.1093/jnci/djk111. PubMed PMID: 17405998.

7. Schairer C, Hisada M, Chen BE, Brown LM, Howard R, Fosså SD, et al. Comparative mortality for 621 second cancers in 29356 testicular cancer survivors and 12420 matched first cancers. Journal of the National Cancer Institute. 2007;99:1248-56. doi: 10.1093/jnci/djm081. PubMed PMID: 17686826.

8. Robinson D, Møller H, Horwich A. Mortality and incidence of second cancers following treatment for testicular cancer. British journal of cancer. 2007;96:529-33. doi: 10.1038/sj.bjc.6603589. PubMed PMID: 17262080.

9. Powles T, Robinson D, Shamash J, Moller H, Tranter N, Oliver T. The long-term risks of adjuvant carboplatin treatment for stage I seminoma of the testis. Annals of Oncology. 2008;19:443-7. doi: 10.1093/annonc/mdm540. PubMed PMID: 18048383.

10. Alanee S, Russo P. Suicide in men with testis cancer. European journal of cancer care. 2012;21:817-21. doi: 10.1111/j.1365-2354.2012.01366.x. PubMed PMID: 22624649.

11. Beard CJ, Travis LB, Chen M-H, Arvold ND, Nguyen PL, Martin NE, et al. Outcomes in stage I testicular seminoma: a population-based study of 9193 patients. Cancer. 2013;119:2771-7. doi: 10.1002/cncr.28086. PubMed PMID: 23633409.

12. Horwich A, Fossa SD, Huddart R, Dearnaley DP, Stenning S, Aresu M, et al. Second cancer risk and mortality in men treated with radiotherapy for stage I seminoma. British journal of cancer. 2014;110:256-63. doi: 10.1038/bjc.2013.551. PubMed PMID: 24263066.

13. Gandaglia G, Becker A, Trinh Q, Abdollah F, Schiffmann J, Roghmann F, et al. Long-term survival in patients with germ cell testicular cancer: a population-based competing-risks regression analysis. European journal of surgical oncology : the journal of the European Society of Surgical Oncology and the British Association of Surgical Oncology. 2014;40:103-12. doi: 10.1016/j.ejso.2013.09.019. PubMed PMID: 24099755.

14. Fung C, Fossa SD, Milano MT, Sahasrabudhe DM, Peterson DR, Travis LB. Cardiovascular Disease Mortality After Chemotherapy or Surgery for Testicular Nonseminoma: A Population-Based Study. Journal of Clinical Oncology. 2015;33:3105-15. doi: 10.1200/JCO.2014.60.3654.

15. Kier MG, Hansen MK, Lauritsen J, Mortensen MS, Bandak M, Agerbaek M, et al. Second Malignant Neoplasms and Cause of Death in Patients With Germ Cell Cancer: A Danish Nationwide Cohort Study. JAMA Oncol. 2016;2(12):1624-7. Epub 2016/10/07. doi: 10.1001/jamaoncol.2016.3651. PubMed PMID: 27711914.

16. Gunnes MW, Lie RT, Bjorge T, Ghaderi S, Syse A, Ruud E, et al. Suicide and violent deaths in survivors of cancer in childhood, adolescence and young adulthood-A national cohort study. Int J Cancer. 2017;140(3):575-80. Epub 2016/10/27. doi: 10.1002/ijc.30474. PubMed PMID: 27750385.

17. Patel HD, Srivastava A, Alam R, Joice GA, Schwen ZR, Semerjian A, et al. Radiotherapy for stage I and II testicular seminomas: Secondary malignancies and survival. Urol Oncol. 2017;35(10):606 e1- e7. Epub 2017/07/18. doi: 10.1016/j.urolonc.2017.06.051. PubMed PMID: 28712791.

18. Zhang L, Hemminki O, Chen T, Yu H, Zheng G, Chattopadhyay S, et al. Second cancers and causes of death in patients with testicular cancer in Sweden. PLoS One. 2019;14(3):e0214410. Epub 2019/03/29. doi: 10.1371/journal.pone.0214410. PubMed PMID: 30921367; PubMed Central PMCID: PMCPMC6438485.
